# The acetylglucosaminyltransferase GnT-Ⅲ regulates erythroid differentiation through ERK/MAPK signaling

**DOI:** 10.1016/j.jbc.2024.108010

**Published:** 2024-11-19

**Authors:** Tiangui Wu, Yuhan Sun, Dan Wang, Tomoya Isaji, Tomohiko Fukuda, Chiharu Suzuki, Hisatoshi Hanamatsu, Takashi Nishikaze, Hiroki Tsumoto, Yuri Miura, Jun-ichi Furukawa, Jianguo Gu

**Affiliations:** 1 Division of Regulatory Glycobiology, Institute of Molecular Biomembrane and Glycobiology, Tohoku Medical and Pharmaceutical University, Sendai, Miyagi, Japan; 2 Division of Glyco-Systems Biology, Institute for Glyco-Core Research, Tokai National Higher Education and Research System, Nagoya, Japan; 3 Solutions COE, Analytical & Measuring Instruments Division, Shimadzu Corporation, Kyoto, Japan; 4 Research Team for Mechanism of Aging, Tokyo Metropolitan Institute for Geriatrics and Gerontology, Tokyo, Japan; 5 Department of Orthopedic Surgery, Hokkaido University Graduate School of Medicine, Sapporo, Japan

**Keywords:** bisected *N*-glycans, chronic myelogenous leukemia, ERK signaling, erythroid differentiation, *N*-glycosylation

## Abstract

Differentiation therapy is an alternative strategy used in treating chronic myelogenous leukemia to induce the differentiation of immature or cancerous cells toward mature cells and inhibit tumor cell proliferation. We aimed to explore *N*-glycans' roles in erythroid differentiation using the sodium butyrate (NaBu)-induced model of K562 cells (WT/NaBu cells). Here, using lectin blot, flow cytometry, real-time PCR, and mass spectrometry analyses, we demonstrated that the mRNA levels of *N*-acetylglucosaminyltransferase Ⅲ ((encoded by the *MGAT3* gene) and its product (bisected *N*-glycans) were significantly increased during erythroid differentiation. To address the importance of GnT*N*-acetylglucosaminyltransferase-Ⅲ in this progress, we established a stable *MGAT3* KO K562 cell line using the CRISPR/Cas9 technology. Compared to WT/NaBu cells, *MGAT3* KO significantly impeded the progression of erythroid differentiation, as shown in decreased cell color and levels of erythroid markers, glycophorin A (CD235a), and β-globin. Consistently, *MGAT3* KO mitigated the inhibitory impact of NaBu on cell proliferation. During induction, *MGAT3* KO suppressed the cellular phosphorylated tyrosine and phospho-extracellular signal–regulated kinase (ERK)1/2 levels. Inhibition of the ERK/mitogen-activated protein kinase signaling pathway using U0126 blocked erythroid differentiation while concurrently suppressing the expression levels of *MGAT3* and bisected *N*-glycans. Furthermore, the lack of bisecting GlcNAc modification on c-Kit and transferrin receptor 1 (CD71) suppressed cellular signaling and accelerated the degradation of the CD71 protein, respectively. Our study highlights the critical role of *MGAT3* in regulating erythroid differentiation associated with the ERK/mitogen-activated protein kinase signaling pathway, which may shed light on identifying new differentiation therapy in chronic myelogenous leukemia.

Chronic myelogenous leukemia (CML) is a clonal myeloproliferative neoplasm originating from a transformed hematopoietic stem cell. This abnormality leads to the emergence of a significant population of clones that proliferate extensively but are unable to undergo complete self-renewal or differentiation (1). This disease is characterized by a reciprocal chromosomal translocation referred to as t (2, 3), resulting in the formation of the Philadelphia chromosome harboring the BCR-ABL gene (4). Currently, molecular target therapies that focus on the ABL1 tyrosine kinase, particularly the application of tyrosine kinase inhibitors, have significantly enhanced the life expectancy of patients with CML and markedly improved their quality of life (5, 6). Unfortunately, the long-term use of tyrosine kinase inhibitors can lead to adverse effects in some patients, and also impose a substantial financial burden (7, 8). In recent years, there has been a growing interest in the potential of inducing differentiation in leukemia cells as a method for achieving disease remission, with differentiation therapy emerging as a promising complementary or alternative approach (9). This therapeutic strategy seeks to promote the maturation and specialization of immature or malignant cells, thereby enabling them to acquire characteristics akin to those of normal cells (10). A notable example of this approach is the differentiation therapy used in acute promyelocytic leukemia (APL), which employs retinoic acid and arsenic trioxide (2).

K562, a CML cell line, is a typical model for investigating erythroid differentiation using sodium butyrate (NaBu) to explore the mechanism of differentiation therapy (11). The induction of erythroid differentiation is a complex and highly orchestrated process. Erythrocytes originate from hematopoietic stem cells *via* a well-defined, sequential differentiation pathway (12). The induction of specific proteins, such as hemoglobin and transferrin receptor 1, also called CD71 (encoded by *TFRC* gene), and activation of signaling pathways are critical in this progression. Consequently, CD71 plays a vital role in erythropoiesis by assisting in regulating iron homeostasis and promoting the development and proper functioning of red blood cells (13). Activation of multiple pathways, particularly the extracellular signal–regulated kinase/mitogen-activated protein kinase (ERK/MAPK) pathway, is crucial in erythroid differentiation. Studies on primary human erythroid progenitor cells have shown that activation of the RAS/ERK pathway is necessary for effective erythropoietin–dependent erythroblast formation (14). Notably, many erythroid markers are glycosylated proteins, and *N*-glycosylation plays a crucial role in regulating pathways (15, 16, 17).

*N-*glycosylation is a prevalent posttranslational modification in mammalian cells (18, 19). Alterations in glycosylation play a critical role in tumor progression and are considered a hallmark of cancer (20). *N*-acetylglucosaminyltransferase Ⅲ (GnT-Ⅲ), encoded by the *MGAT3* gene, is a crucial glycosyltransferase known for synthesizing a bisecting *N*-acetylglucosamine residue and has gained attention for its role in physiology (21) and tumor biology (3, 22). For instance, *MGAT3* overexpression suppresses TGF-β–induced epithelial-mesenchymal transition (EMT) by prolonging E-cadherin turnover and inhibiting the formation of the β-catenin/p-Smad complex (23). Additionally, GnT-Ⅲ negatively regulates chemoresistance by suppressing P-glycoprotein expression *via* the TNFR2-NF/κB signaling pathway (3). These findings underscore GnT-Ⅲ's significant role in modulating cancer progression and resistance mechanisms. In addition, some studies show that *N*-glycosylation is associated with cell differentiation. For instance, overexpression of α-1,6-fucosyltransferase suppresses hemoglobin production in both murine and human erythroleukemia (HEL) cells, while its downregulation promotes erythroid differentiation and hemoglobin production (24). These studies highlight the intricate roles of *N*-glycosylation in tumor initiation, progression, and differentiation. However, mechanisms linking *N*-glycosylation to differentiation remain understudied, warranting further investigation.

In this study, we utilized a conventional differentiation model employing NaBu treatment (WT/NaBu cells) and observed a notable increase in the expression levels of *MGAT3* and its product, bisected *N*-glycans, during the differentiation process. KO of *MGAT3* inhibited this differentiation progression, correlating with reduced levels of erythroid markers such as glycophorin A (CD235a, encoded by *GYPA* gene) and β-globin. Importantly, treatment with U0126, a specific MEK inhibitor, significantly suppressed the differentiation and *MGAT3* expression. These findings uncover a novel mechanism underlying cell differentiation, demonstrating that GnT-Ⅲ plays a crucial role in promoting erythroid differentiation. The induction of GnT-Ⅲ or ERK suggests potential strategies to enhance the efficacy of differentiation therapy, potentially offering new avenues for therapeutic intervention in conditions like CML where differentiation defects are implicated.

## Results

### NaBu-induced differentiation toward the erythroid lineage in K562 cells

**Figure 1:**
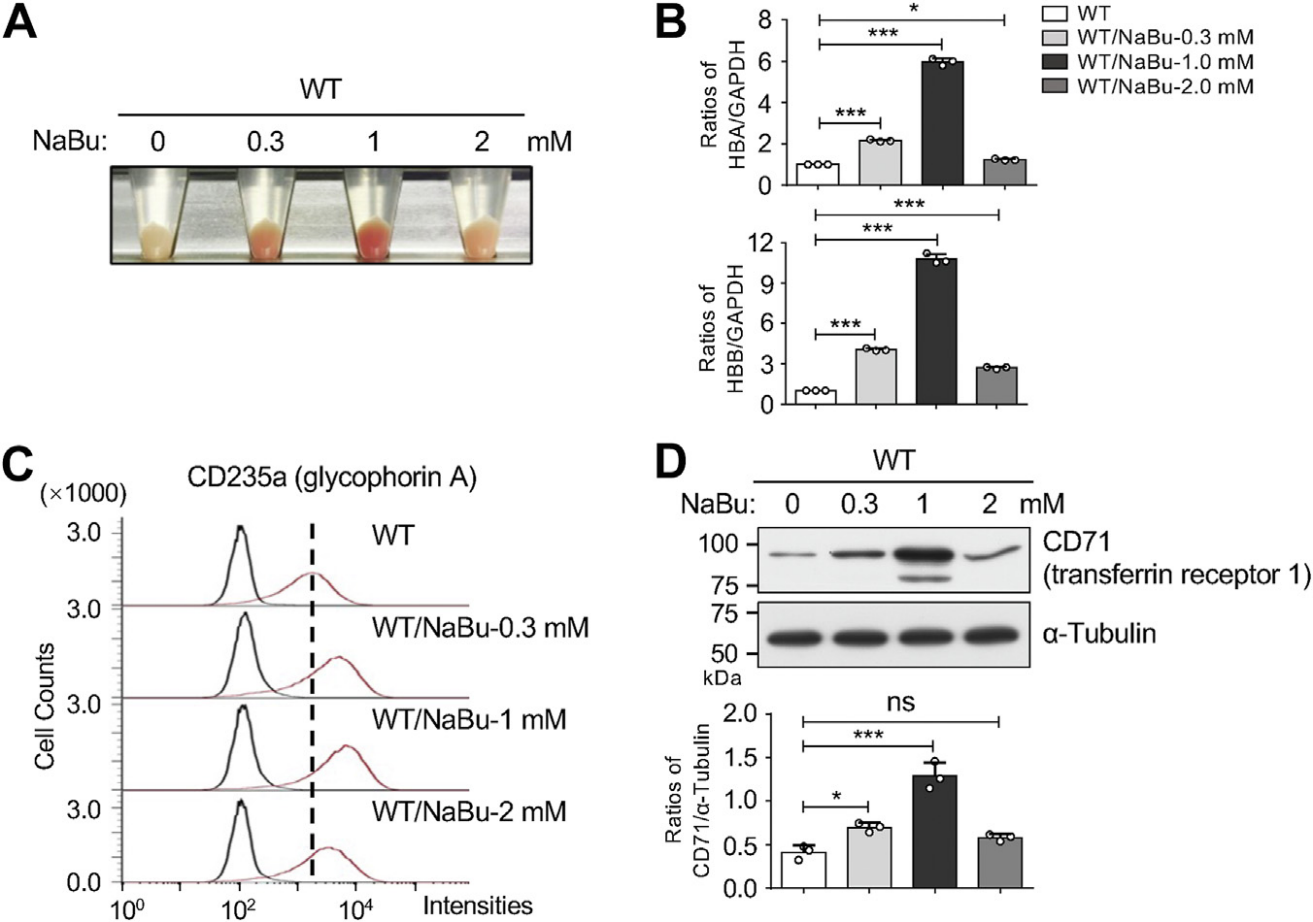
**NaBu induced differentiation of K562 cells toward the erythroid lineage**. *A*, K562 cells were cultured with varying concentrations of NaBu (0, 0.3, 1, 2 mM) for 96 h, and cells treated with 1 mM NaBu appeared significantly *reddish*. *B*, mRNA levels of the *HBA* and *HBB* were detected by qPCR, with GAPDH used as an internal control. The intensity ratio of WT was set as 1.0. Data are presented as mean ± SD from three independent experiments. *p* values were calculated using one-way ANOVA. ∗*p* < 0.05 and ∗∗∗*p* < 0.001. *C*, protein levels of CD235a on the cell surface after treatment with varying concentrations of NaBu were analyzed by flow cytometry using an anti-CD235a antibody. *D*, equal amounts of cell lysates were subjected to Western blotting using an anti-CD71 antibody to assess CD71 expression, with α-Tubulin serving as a loading control. Data were quantified using Image J software (https://imagej.net/ij/) and obtained from three independent experiments. All values reflect one-way ANOVA with Tukey’s *post hoc* analysis as the mean ± SD. ∗*p* < 0.05; ∗∗∗*p* < 0.001; and ns, no significance. NaBu, sodium butyrate.

In this study, we induced differentiation of HEL K562 cells towards the erythroid lineage using NaBu, a well-established inducer (25). This differentiation process is characterized by changes in cell color, increased production of hemoglobin (specifically α-globin and β-globin), and the acquisition of erythrocyte surface markers such as CD235a and CD71 (26). Here, we cultured K562 cells with different concentrations of NaBu for 96 h to assess its efficacy in inducing erythroid differentiation. Visual observation of cell color changes indicated that NaBu at 1 mM was the most effective concentration for inducing differentiation in WT/NaBu cells (Fig. 1*A*). Quantitative PCR (qPCR) analysis revealed significant increases in the mRNA levels of α-globin and β-globin following NaBu treatment, particularly at 1 mM (Fig. 1*B*). Similarly, flow cytometry (Fig. 1*C*) and Western blotting analysis (Fig. 1*D*) confirmed significant upregulation of CD235a and CD71 protein expression upon treatment with NaBu 1 mM. These findings strongly indicate that NaBu at a concentration of 1 mM effectively induces erythroid differentiation in K562 cells, as evidenced by changes in cell morphology, hemoglobin production, and expression of erythrocyte surface markers.

### Bisected *N*-glycans were increased in WT/NaBu cells

**Figure 2:**
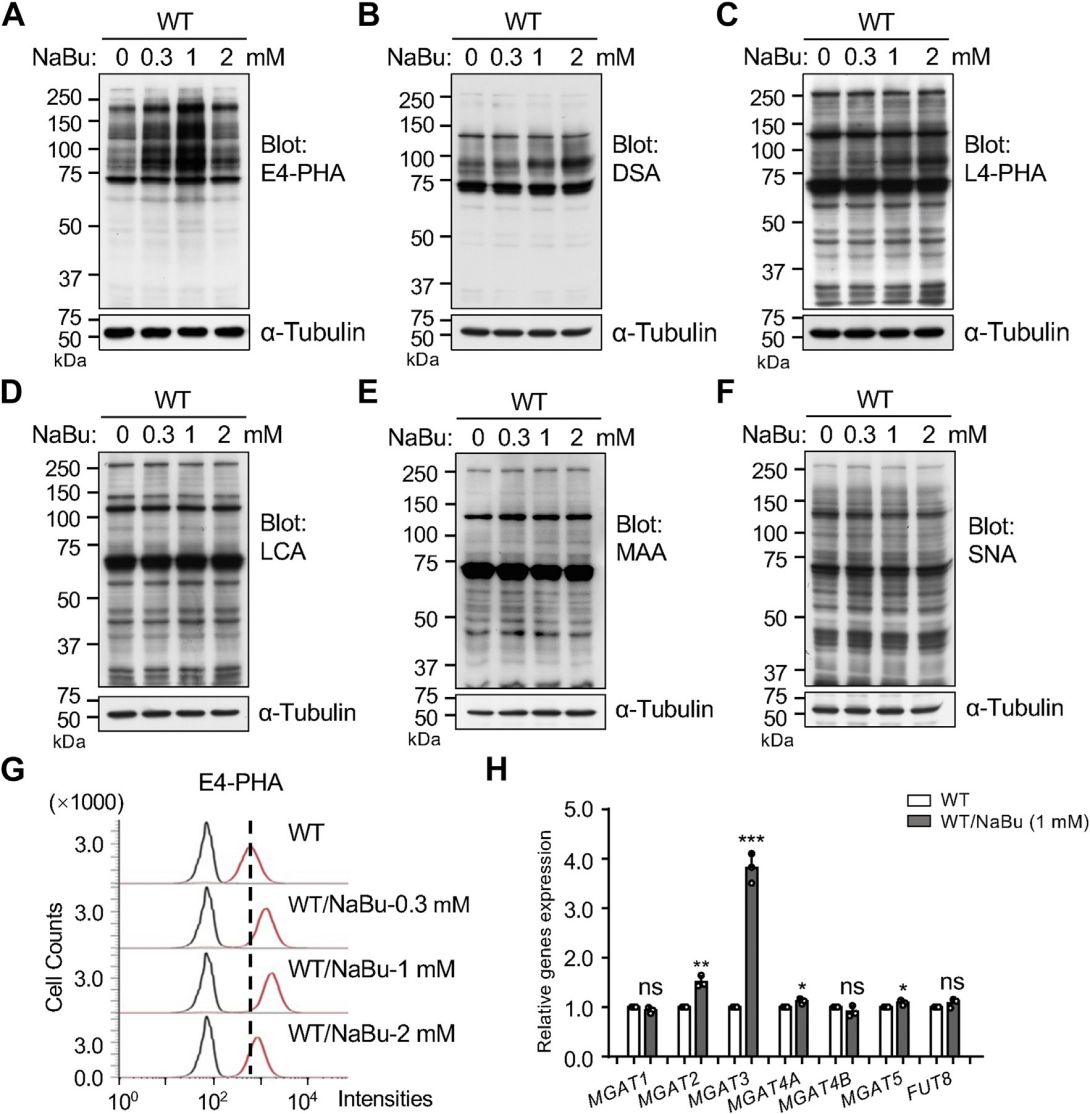
**Comparison of the expression levels of *N*-glycans and related glycosyltransferases between WT and WT/NaBu cells**. The same amounts of cell lysates from WT and NaBu-induced K562 cells were subjected to 7.5% SDS-PAGE gel and stained with various lectins to assess glycosylation changes, such as E4-PHA (*A*), DSA (*B*), L4-PHA (*C*), LCA (*D*), MAA (*E*), and SNA (*F*) lectins, which preferentially recognizes the bisecting GlcNAc, GlcNAcβ1,4 mannosyl-, GlcNAcβ1,6 mannosyl-, α1,6 fucose, α2,3 sialylated, and α2,6 sialylated *N*-glycans, respectively. α-Tubulin was used as a loading control. *G*, expression levels of bisected *N*-glycans on the cell surface were detected by flow cytometry using E4-PHA lectin. *H*, mRNA levels of several *N*-acetylglucosaminyltransferases involved in the synthesis of GlcNAc branched *N*-glycans were determined using qPCR. GAPDH was used as an internal control, and values were normalized to WT cells without NaBu (set as 1.0). Statistical significance was assessed using the unpaired Student’s *t* test, with *p* values indicated as ∗*p* < 0.05; ∗∗*p* < 0.01; ∗∗∗*p* < 0.001, or denoted as no significance (ns). DSA, *Datura stramonium* agglutinin; E4-PHA, *Phaseolus vulgaris* erythroagglutinin, LCA, *Lens culinaris* agglutinin; MAA, *M*. *amurensis*; NaBu, sodium butyrate; qPCR, quantitative PCR; SNA, *S*. *nigra* agglutinin.

In our comparative analysis of *N*-glycans expression patterns between WT and WT/NaBu cells, we employed six distinct lectins: *Phaseolus vulgaris* erythroagglutinin (E4-PHA), *Datura stramonium* agglutinin (DSA), *Phaseolus vulgaris* leucoagglutinin (L4-PHA), *Lens culinaris* agglutinin (LCA), *M*. *amurensis* (MAA), and *S*. *nigra agglutinin* (SNA). Each lectin preferentially recognizes specific glycan structures: E4-PHA recognizes bisected *N*-glycans, DSA binds to β1,4-GlcNAc-branched *N*-glycans, L4-PHA recognizes β1,6-GlcNAc-branched *N*-glycans, LCA detects α1,6-linked fucose, and MAA and SNA recognize α2,3 sialylation and α2,6 sialylation, respectively (27). Lectin blot analysis revealed a significant increase in reactivity with E4-PHA in WT/NaBu cells compared to WT cells, particularly evident at NaBu concentration of 1 mM, which corresponds with the observed erythroid differentiation (Fig. 2*A*). Conversely, no significant changes were observed in the other lectin blots)—such as DSA (Fig. 2*B*), L4-PHA (Fig. 2*C*), LCA (Fig. 2*D*), MAA (Fig. 2*E*), and SNA (Fig. 2*F*)—between WT and WT/NaBu cells. These bands, mobility at approximately 70 kDa and 130 kDa, were still observed in all types of lectins after removal *N*-glycans using peptide-*N*-glycosidase F (PNGase F), including the ABC kit without a lectin (Fig. S1), suggesting they represent nonspecific staining. The increased reactivity with E4-PHA in WT/NaBu cells was further confirmed by flow cytometry (Fig. 2*G*). Additionally, qPCR analysis showed a significant increase in the mRNA level of *MGAT3* in WT/NaBu cells compared to WT cells, corroborating the lectin blot results (Fig. 2*H*).

Given that NaBu acts as a histone deacetylase inhibitor, we employed another histone deacetylase inhibitor, suberanilohydroxamic acid (SAHA), to investigate the relationship between histone acetylation and *MGAT3* expression. As shown in Fig. S2, SAHA did not induce erythroid differentiation in K562 cells (as indicated by the lack of reddish color). In addition, results from lectin blot and flow cytometry analysis using E4-PHA, along with qPCR, demonstrated that the levels of bisected *N*-glycans and *MGAT3* expression remained unchanged with SAHA treatment. Conversely, Frank CL *et al*. reported that NaBu and SAHA exhibited similar effects on chromatin accessibility in K562 cells (28). These findings suggest that NaBu upregulates GnT-Ⅲ independently of histone acetylation.

To further investigate whether the phenomenon is consistent during NaBu induction, we employed another widely recognized erythroid differentiation inducer, hemin, to establish a hemin-induced erythroid differentiation model in K562 cells. Consistently, levels of bisected *N*-glycans and *MGAT3* expression were significantly increased during this induction (Fig. S3). Furthermore, we selected another cell line, HEL cells, to test this phenomenon. The results indicated that hemin could also induce erythroid differentiation in HEL cells, with significant upregulation of bisected *N*-glycans and *MGAT3* during the process (Fig. S3). These results suggest GnT-Ⅲ can be upregulated in erythroid differentiation.

**Figure 3:**
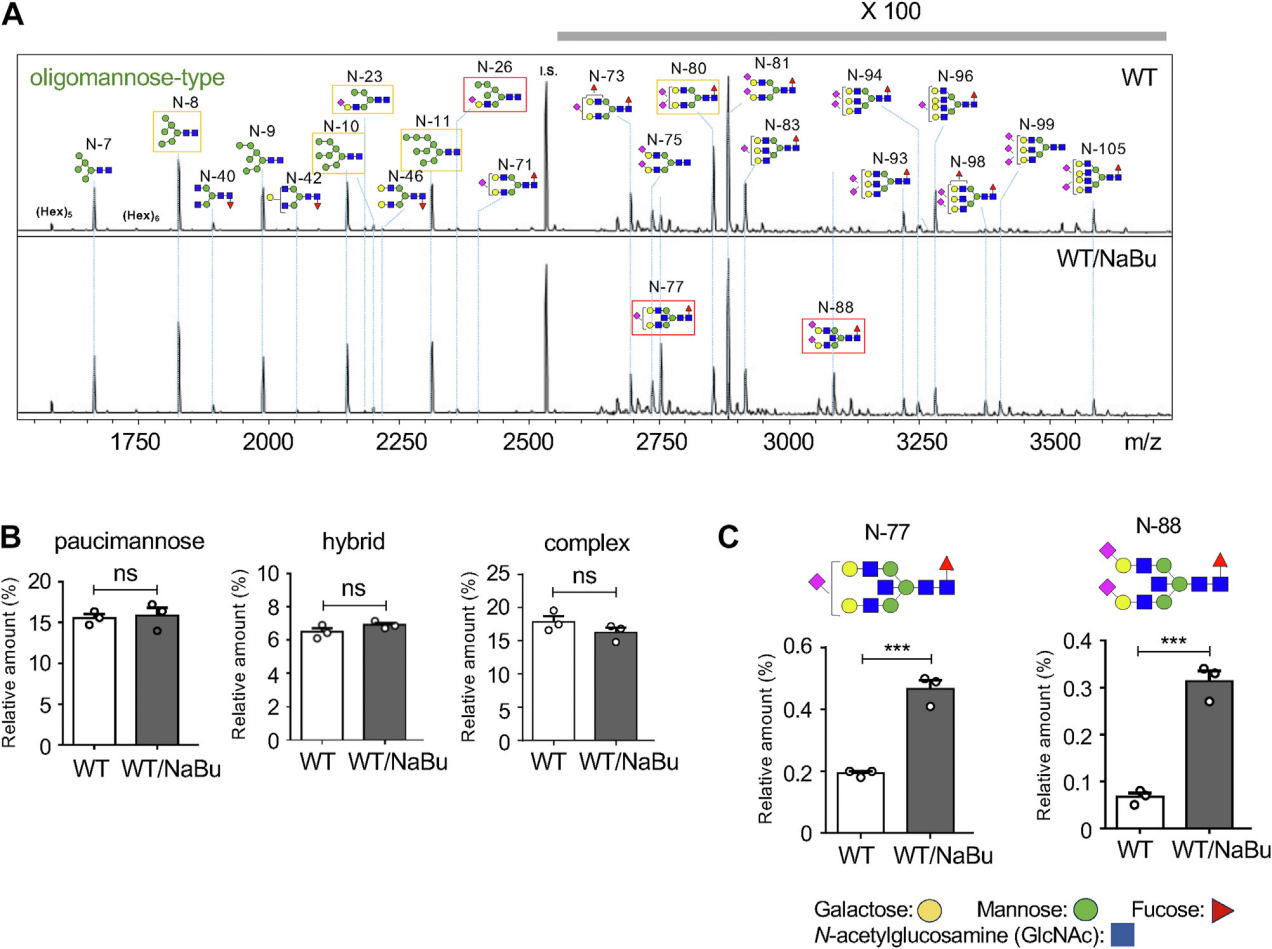
**MALDI-TOF MS analysis of *N*-glycans in K562 and K562/NaBu cells**. *A*, MALDI-TOF MS spectra of *N*-glycans from K562 and K562/NaBu cells were shown. To highlight differences in expression levels, the region from *m/z* over 2600 to 3750 was magnified 100-fold. *B*, the relative expression levels of three types of *N*-glycans (paucimannose, hybrid, and complex) were compared. The total expression level of oligomannose-type in each cell was set as 100% for comparison. *C*, the relative expression levels of bisected *N*-glycans were compared between K562 and K562/NaBu cells. Data was acquired from three independent experiments using the unpaired Student’s *t* test as mean ± SD. ∗∗∗*p* < 0.001; ns, no significance. MALDI-TOF MS, matrix-assisted laser desorption/ionization time-of-flight mass spectrometry; NaBu, sodium butyrate.

To validate the changes in the *N*-glycan structures as described above, we conducted matrix-assisted laser desorption/ionization time-of-flight mass spectrometry (MALDI-TOF MS) analysis, which revealed 108 distinct *N*-glycans in WT and WT/NaBu cells (Table S1). The spectra of major *N*-glycans are shown in Fig. 3*A*. While the relative amounts of *N*-glycan types (paucimannose-, hybrid-, and complex-type) did not show significant differences (Fig. 3*B*), two signals of *N*-77 and *N*-88 were notably increased in WT/NaBu cells (Fig. 3*C*). Furthermore, we confirmed that bisecting GlcNAc was included in both *N*-glycans (*N*-77 and *N*-88) by LC-tandem mass spectrometry (MS/MS) analysis as shown in Fig. S4. In addition, putative bisected *N*-glycans from glycan compositions were summarized in Table S2. The expression of most bisected *N*-glycans was increased in WT/NaBu cells. This finding aligns with the lectin blot results and provides robust evidence that NaBu-induced erythroid differentiation is associated with upregulated expression of GnT-Ⅲ and its product, bisected *N*-glycans.

### *MGAT3* KO inhibited the erythroid differentiation induced by NaBu in K562 cells

**Figure 4:**
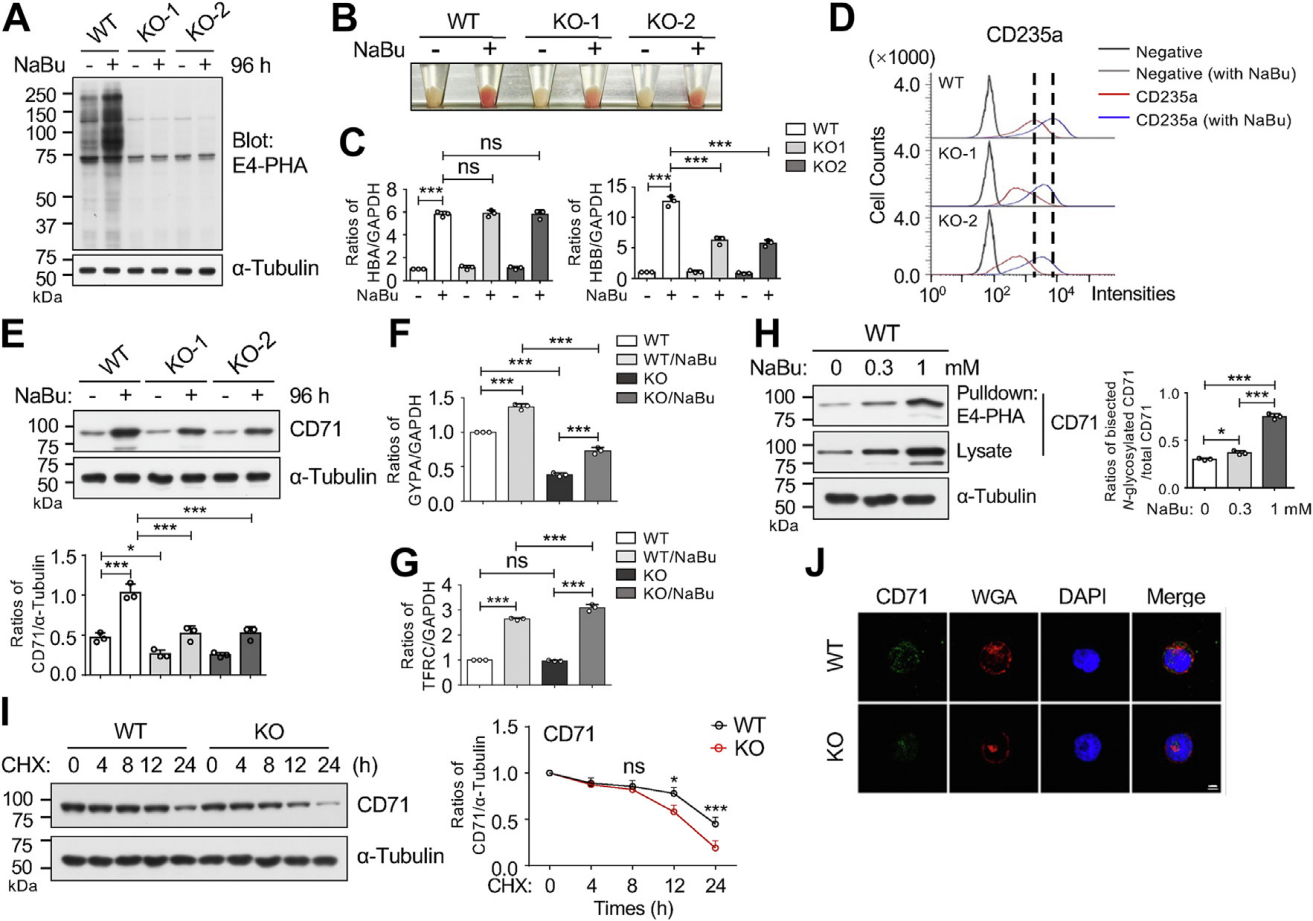
**Effects of GnT-Ⅲ on NaBu-induced erythroid differentiation**. *A*, *MGAT3* KO K562 cell line was established by using CRISPR/Cas9 technology. Equal amounts of proteins from WT and two *MGAT3* KO cell lines, with or without NaBu treatment, were subjected to lectin blotting using E4-PHA. α-Tubulin was used as a loading control. *B*, coloration was compared between WT and *MGAT3* KO cells after treatment with 1 mM NaBu for 96 h. *C*, mRNA expression levels of *HBA* and *HBB* in WT and two *MGAT3* KO cell lines, with or without NaBu treatment, were compared using qPCR. GAPDH served as the internal control. All values were normalized to the GAPDH levels, with the ratio of WT without NaBu set as 1.0. Data are presented as the mean ± SD from three independent experiments. ∗∗∗*p* < 0.001; ns, no significance. *D*, expression levels of CD235a protein on the cell surface were analyzed by flow cytometry. *E*, expression levels of CD71 were analyzed by Western blotting. α-Tubulin was used as a loading control. The results are presented as the mean ± SD from three independent experiments. ∗*p* < 0.05 and ∗∗∗*p* < 0.001. mRNA expression levels of *GYPA* (*F*) and *TFRC* (*G*) in WT and *MGAT3* KO-1 cells, with or without NaBu treatment, were compared using qPCR. GAPDH served as the internal control. All values were normalized to the GAPDH levels, with the ratio of WT without NaBu set as 1.0. Data are presented as mean ± SD from three independent experiments using one-way ANOVA with Tukey’s *post hoc* analysis. ∗∗∗*p* < 0.001; ns, no significance. *H*, equal amounts of proteins were pulled down with E4-PHA-agarose, and the samples or cell lysates were subjected to Western blotting with an anti-CD71 antibody. α-Tubulin was used as a loading control. The relative ratios of CD71 containing bisecting GlcNAc were normalized to the total CD71 levels at each point. Data were quantified using ImageJ software and derived from three independent experiments. All values reflect one-way ANOVA with Tukey’s *post hoc* analysis as the mean ± SD. *∗p* < 0.05 and ∗∗∗*p* < 0.001. *I*, WT and *MGAT3* KO cells were cultured with or without 50 mM cycloheximide (CHX), a protein synthesis inhibitor, for indicated times (0, 4, 8, 12, and 24 h). Equal amounts of proteins were subjected to Western blotting with anti-CD71 antibodies. α-Tubulin was used as a loading control. Relative ratios were calculated based on the density of CD71 relative to α-Tubulin at each indicated time point, with the ratio at 0 h (without CHX) set as 1.0. Values represent mean ± SD from three independent experiments. ∗*p* < 0.05; ∗∗∗*p* < 0.001; and ns, no significance. *J*, CD71 protein expression in WT and *MGAT3* KO cells was detected using anti-CD71 antibody (*green*). The plasma membrane was stained using WGA lectin (*red*). Detection was accomplished using a ZEISS LSM 900 confocal microscope a ZEISS LSM 900 confocal microscope cells. Scale bars represent 10 μm. E4-PHA, *Phaseolus vulgaris* erythroagglutinin; NaBu, sodium butyrate; qPCR, quantitative PCR; WGA, Wheat germ agglutinin.

To investigate the role of GnT-Ⅲ in this process, we generated an *MGAT3* KO (*MGAT3* KO) K562 cell line using CRISPR/Cas9 technology. Subsequently, we randomly selected two KO single-cell lines confirmed by genomic sequence analysis (Fig. S5). As anticipated, both WT and WT/NaBu *MGAT3* KO cells exhibited nearly abolished reactivity with E4-PHA lectin (Fig. 4*A*). In comparison to WT cells, *MGAT3* KO cells showed significant impairment in cellular differentiation induced by NaBu (Fig. 4*B*). We further investigated changes in markers associated with erythroid differentiation. qPCR analysis revealed that NaBu induction significantly upregulated α-globin and β-globin mRNA levels in both WT and *MGAT3* KO cells (Fig. 4*C*). However, *MGAT3* KO specifically suppressed the induction of β-globin mRNA levels, while α-globin mRNA levels remained unaffected in *MGAT3* KO cells compared to WT/NaBu cells (Fig. 4*C*). The expression levels of CD235a protein on the cell surface (Fig. 4*D*) and CD71 protein in cell lysates (Fig. 4*E*) were downregulated in *MGAT3* KO cells compared the corresponding WT cells.

Since both KO cells showed similar phenotypes as described above, we selected KO-1 for the following studies. Similar to *HBB* mRNA expression, as shown in Fig. 4*C*, *MGAT3* KO also suppressed the induction of *GYPA* mRNA (Fig. 4*F*). Surprisingly, although NaBu induction significantly increased both *TFRC* mRNA (Fig. 4*G*) and CD71 protein levels (Fig. 4*E*) in both WT and *MGAT3* KO cells, *MGAT3* KO specifically suppressed the induction for CD71 protein levels, but not CD71 mRNA levels, in *MGAT3* KO cells compared to the WT/NaBu cells (Fig. 4, *E* and *G*). CD71 is known to be a heavily *N*-glycosylated membrane protein (16, 29). As shown in Fig. 4*H*, the pulldown assay using E4-PHA indicated that CD71 protein underwent bisecting GlcNAc modification upon NaBu induction. The ratio of bisected *N*-glycosylated CD71 to total CD71 was also increased during the induction. To elucidate the underlying molecular mechanism for the decreased CD71 protein levels in *MGAT3* KO cells, we employed cycloheximide, a protein synthesis inhibitor, to assess CD71 protein stability. The decay rates of CD71 protein in *MGAT3* KO cells were significantly accelerated compared to WT cells (Fig. 4*I*). Subsequently, the intracellular localization of the CD71 protein was observed using immunofluorescence. As shown in Fig. 4*J*, the staining of CD71 on the cell surface was lower in *MGAT3* KO cells than in WT cells. These results suggest that *MGAT3* KO suppresses CD71 expression on the cell surface.

### *MGAT3* KO efficiently increased cell proliferation during NaBu-induced differentiation

**Figure 5:**
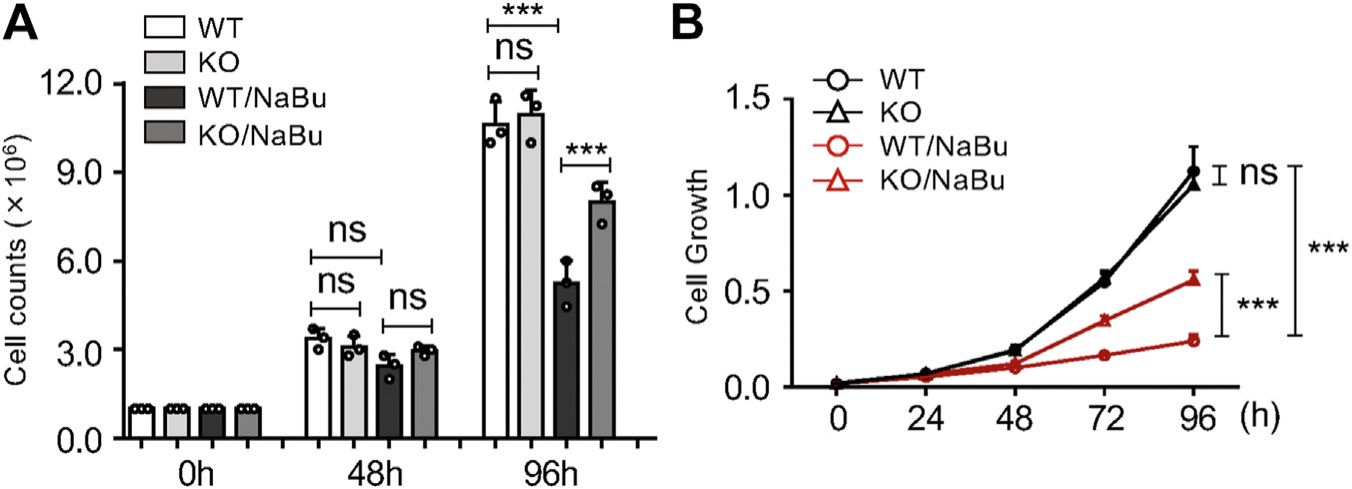
***MGAT3* KO resulted in increased cell proliferation during NaBu-induced differentiation**. *A*, WT and *MGAT3* KO cells were cultured with or without NaBu treatment for indicated times, and then cell numbers were evaluated using a cell counter (ERMA). The results are presented as the mean ± SD from three independent experiments using one-way ANOVA with Tukey’s *post hoc* analysis. ∗∗∗*p* < 0.001; ns, no significance. *B*, WT and *MGAT3* KO cells were seeded in 96-well plates and cultured with 1 mM NaBu for indicated times. Cell proliferation was measured *via* Cell Counting Kit-8 assay as described in “Experimental procedures.” Data are present as the mean ± SD (n = 3), two-way ANOVA. ∗∗∗*p* < 0.001; ns, no significance. NaBu, sodium butyrate.

It is widely recognized that there exists a notable inverse relationship between cell proliferation and differentiation (30). Given that *MGAT3* KO markedly suppressed NaBu-induced differentiation, as previously described, we further investigated its impact on cell proliferation. While *MGAT3* KO did not exhibit a significant effect on basal cell proliferation, it hindered the suppression of cell proliferation, as validated by cell counts (Fig. 5*A*) and the Cell Counting Kit-8 (CCK-8) assay (Fig. 5*B*) during NaBu-induced differentiation, further supporting that GnT-Ⅲ does play a crucial role in the differentiation.

### *MGAT3* KO suppressed the activation of the ERK1/2/MAPK signaling pathway in the induction by NaBu

**Figure 6:**
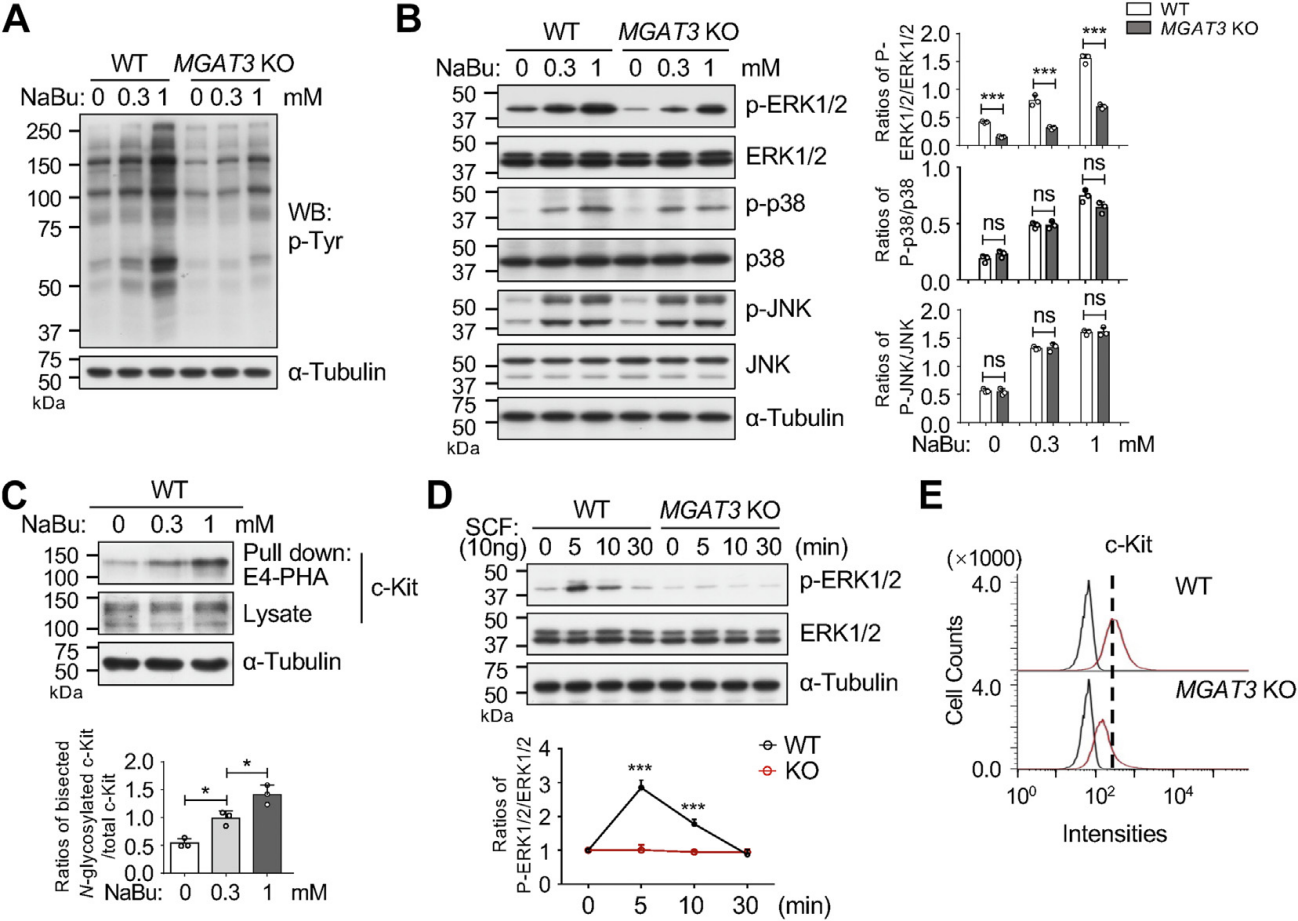
**Effects of *MGAT3* KO on cellular signaling**. WT and *MGAT3* KO cells were cultured with or without NaBu at indicated concentrations for 96 h. *A*, equal amounts of proteins from these cells were subjected to Western blotting using an anti-p-Tyr antibody. α-Tubulin was used as a loading control. *B*, equal amounts of proteins were loaded into a 15% SDS-PAGE gel to compare the expression levels and phosphorylation states of ERK1/2, p38, and JNK in the indicated cells. α-Tubulin was used as a loading control. Relative ratios were calculated based on the density of each phosphorylated form relative to the total form at each indicated time point. Data were quantified using Image J software and obtained from three independent experiments. *p* values were calculated using two-way ANOVA as the mean ± SD. ∗∗∗*p* < 0.001; ns, no significance. *C*, equal amounts of proteins were incubated with E4-PHA-agarose, and the samples were subjected to Western blotting with an anti-c-Kit antibody. α-Tubulin was used as a loading control. The experiments were independently repeated three times. Relative ratios were calculated based on the intensity of c-Kit containing bisecting GlcNAc relative to total c-Kit at each point. All values represent one-way ANOVA with Tukey's *post hoc* analysis as mean ± SD. ∗*p* < 0.05. *D*, effects of SCF stimulation on the ERK1/2 signaling were compared between WT and *MGAT3* KO cells. Equal amounts of proteins were subjected to Western blotting with indicated antibodies. α-Tubulin was used as a loading control. Relative ratios were calculated based on the band intensity of each phosphorylated ERK1/2 relative to the total ERK1/2 at each time point, and the ratio at 0 min for each group was set as 1.0. The experiments were independently repeated three times. *p* values were calculated using two-way ANOVA. ∗∗∗*p* < 0.001. *E*, comparison of c-Kit expression levels on the cell surface in WT and *MGAT3* KO cells using flow cytometry. ERK, extracellular signal–regulated kinase.

To investigate the underlying molecular mechanisms by which GnT-Ⅲ regulates NaBu-induced erythroid differentiation, we examined cellular signaling. Previous studies have shown that the receptor tyrosine kinases (RTKs)/MAPK pathway is one of the most crucial pathways associated with differentiation (31, 32, 33). Here, we first examined the phospho-tyrosine levels to evaluate RTK activation. As shown in Fig. 6*A*, the phospho-tyrosine levels were induced by NaBu in WT cells in a dose-dependent manner, but this induction was greatly inhibited by *MGAT3* KO, regardless of NaBu treatment, compared to the WT cells. Subsequently, downstream signals were further examined. Western blot analysis showed increased phosphorylation levels of ERK1/2, p38, and JNK in both WT and *MGAT3* KO cells (Fig. 6*B*). However, interestingly, *MGAT3* KO suppressed the induction of phospho-ERK1/2 by NaBu in *MGAT3* KO cells compared to the WT cells, while phospho-p38 and phospho-JNK levels remained unaffected. Based on these results, we speculated that the ERK1/2 signal pathway is involved in regulating NaBu-induced differentiation by GnT-Ⅲ.

The c-Kit protein, a crucial member of the RTKs family that mediates ERK1/2 activation (34, 35), plays a significant role in erythropoiesis. The interaction between c-Kit and ligand stem cell factor is essential for this process. Interestingly, the modification by bisecting GlcNAc on c-Kit was significantly upregulated in a dose-dependent manner (Fig. 6*C*), suggesting that c-Kit is one of the target proteins for GnT-Ⅲ during NaBu-induced differentiation. The total c-Kit expression levels in cell lysates were similar among the cells treated with or without NaBu (Fig. 6*C*). Moreover, stimulation with stem cell factor resulted in a significant increase in the ERK pathway activation, which was significantly inhibited in the *MGAT3* KO cells (Fig. 6*D*). Interestingly, the result of the flow cytometry analysis showed the absence of *MGAT3* significantly reduced the expression of c-Kit on the cell surface, compared to WT cell (Fig. 6*E*). Therefore, it can be interpreted that that GnT-Ⅲ–mediated modification of RTKs such as c-Kit may upregulate their expression levels on the cell surface, as well as may enhance ligand–receptor interactions, thereby upregulating ERK activation. The detailed mechanism remains further study.

### U0126, a MEK inhibitor, inhibited NaBu-induced erythroid differentiation in K562 cells

**Figure 7:**
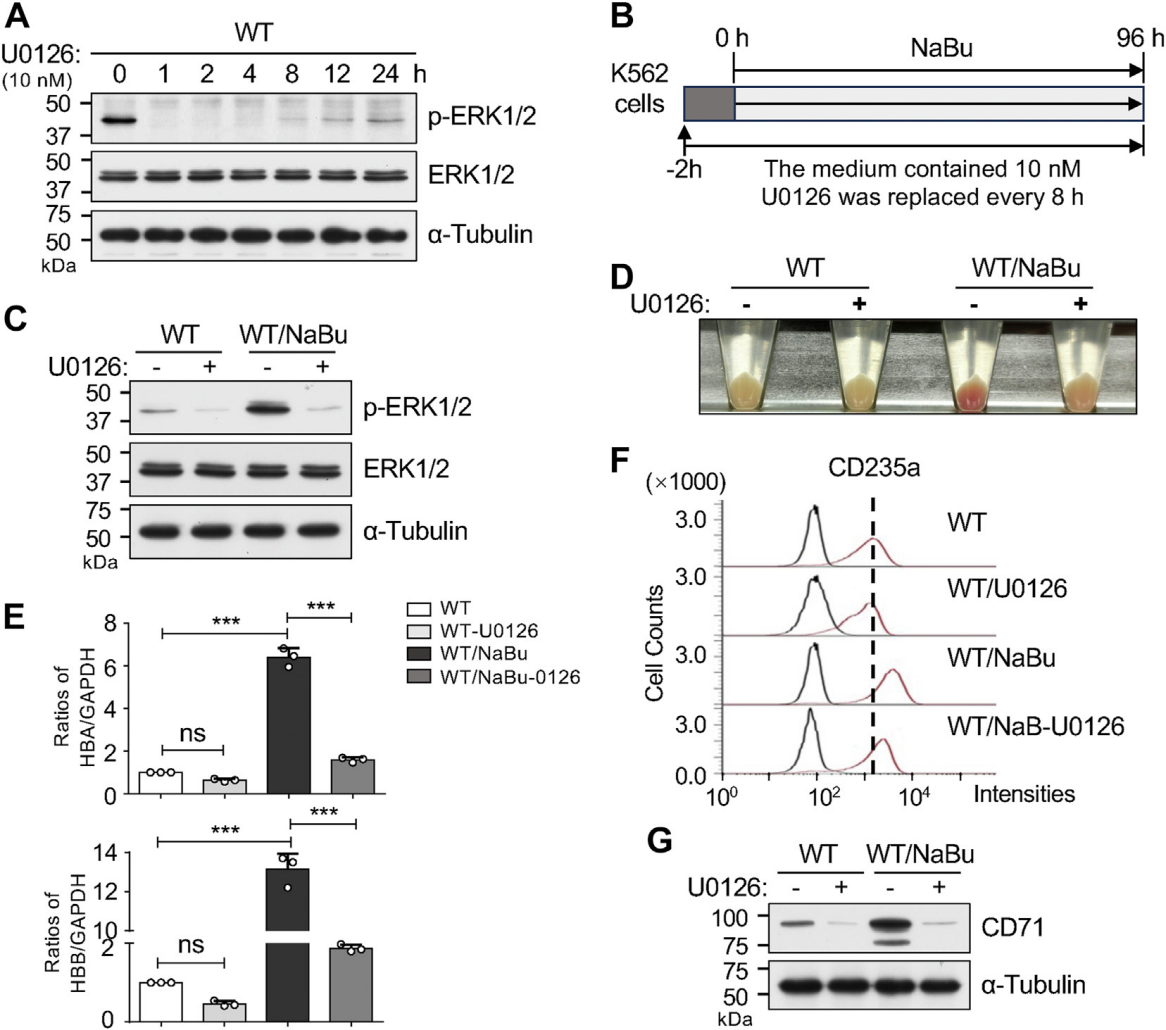
**Inhibition of ERK1/2 signaling suppressed NaBu-induced erythroid differentiation**. *A*, WT K562 cells were cultured with or without 10 nM U0126, a highly selective inhibitor of MEK1/2, for the indicated times (0, 1, 2, 4, 8, 12, and 24 h). Equal amounts of proteins from these cells were subjected to Western blotting using the indicated antibodies. α-Tubulin was used as a loading control. *B*, a schedule depicts inhibition of the ERK1/2 pathway using U0126 during the NaBu-induced erythroid differentiation. *C*, based on the treatment as described in (*B*), equal amounts of cell lysates were subjected to Western blotting using indicated antibodies. α-Tubulin was used as a loading control. *D*, effects of U0126 on coloration in WT cells with or without NaBu treatment. *E*, mRNA expression levels of *HBA* and *HBB* were compared using qPCR in WT and WT/NaBu cells treated with or without U0126. GAPDH served as the internal control. All values were normalized to the GAPDH levels, with the ratio of WT without U0126 set as 1.0. Data are presented as mean ± SD from three independent experiments using one-way ANOVA with Tukey's *post hoc* analysis. ∗∗∗*p* < 0.001; ns, no significance. *F*, expression levels of CD235a on the cell surface were analyzed by flow cytometry using CD235a antibody. *G*, expression levels of CD71 were analyzed by Western blotting. α-Tubulin was used as a loading control. The experiments were independently repeated three times. ERK, extracellular signal–regulated kinase; NaBu, sodium butyrate; qPCR, quantitative PCR.

To demonstrate the importance of the MEK/ERK1/2 pathway in NaBu-induced erythroid differentiation, K562 cells were treated with or without U0126. After incubating K562 cells with U0126 for the indicated times, the phosphorylation levels of ERK1/2 induced by NaBu were effectively suppressed by U0126 as early as 1 h, with a gradual rebound at 8, 12, or 24 h (Fig. 7*A*), suggesting the inhibitory effect of U0126 last for 8 h. Consequently, we replaced the cell culture medium containing fresh U0126 every 8 h during the induction period, as depicted in Fig. 7*B*. As expected, U0126 significantly suppressed the phospho-ERK1/2 levels in both WT cells and WT/NaBu cells (Fig. 7*C*). Concurrently, visual inspection of cell differentiation supported a pronounced inhibition by U0126 of NaBu-induced erythroid differentiation (Fig. 7*D*).

Furthermore, mRNA levels of α-globin and β-globin were significantly decreased after U0126 treatment, as validated by qPCR assay (Fig. 7*E*). Additionally, expression levels of CD235a protein were examined by flow cytometry (Fig. 7*F*), and CD71 protein levels were assessed by Western blotting (Fig. 7*G*). The results showed that U0126 inhibited the expression levels of CD235a protein induced by NaBu (Fig. 7*F*) and suppressed CD71 protein expression in both WT and WT/NaBu cells (Fig. 7*G*). These findings further confirm the critical role of the ERK1/2 signaling pathway in NaBu-induced erythroid differentiation.

### Inhibition of the ERK1/2 signaling downregulated the expression levels of GnT-Ⅲ and bisected *N*-glycans

**Figure 8:**
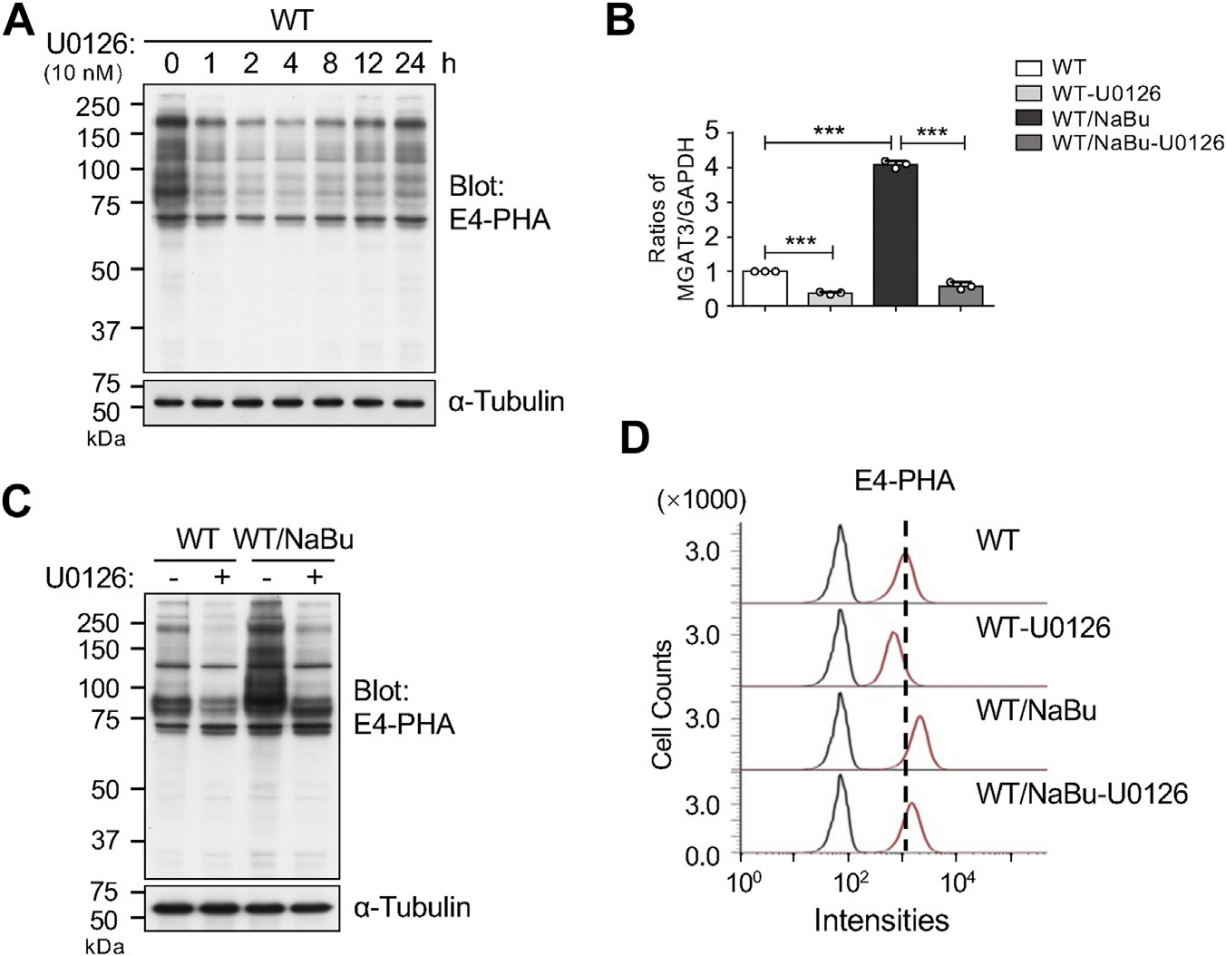
**Expression levels of bisected *N*-glycans and *MGAT3* were significantly decreased after treatment with U0126**. *A*, equal amounts of proteins from K562 WT cells treated with or without U0126 for the indicated times were subjected to lectin blotting using E4-PHA. α-Tubulin was used as a loading control. *B*, mRNA levels of *MGAT3* were measured using qPCR in WT and WT/NaBu cells treated with or without U0126. GAPDH served as the internal control. All values were normalized to the GAPDH levels, with the ratio of WT without U0126 set as 1.0. Data are presented as mean ± SD from three independent experiments. *p* values were calculated using one-way ANOVA with Tukey's *post hoc* analysis. ∗∗∗*p* < 0.001. *C*, effects of U0126 on expression levels of bisected *N*-glycans in cell lysates using E4-PHA lectin blotting in WT and WT/NaBu cells. α-Tubulin was used as a loading control. *D*, effects of U0126 on expression levels of bisected *N*-glycans on the cell surface were analyzed using flow cytometry in WT and WT/NaBu cells. NaBu, sodium butyrate; qPCR, quantitative PCR.

To elucidate the relationship between the ERK1/2 signaling and GnT-Ⅲ, we investigated the impact of inhibiting the ERK1/2 pathway with U0126 on the expression of bisected *N*-glycans. Lectin blot analysis using E4-PHA demonstrated that the levels of bisected *N*-glycans were significantly suppressed as early as 1 h after U0126 treatment, reaching the lowest expression at 2, 4, and 8 h, followed by a rebound observed from 12 h onward (Fig. 8*A*), which paralleled the phospho-ERK1/2 dynamics shown in Fig. 7*A*. This suppression was corroborated by qPCR analysis of *MGAT3* levels (Fig. 8*B*), where U0126 markedly inhibited *MGAT3* expression in both WT cells and WT/NaBu cells. Furthermore, lectin blotting (Fig. 8*C*) and flow cytometry using E4-PHA (Fig. 8*D*) revealed that U0126 downregulated the expression levels of bisected *N*-glycans induced by NaBu. These findings collectively support the idea that the ERK1/2 signaling is crucial for the expression of GnT-Ⅲ and its product, bisected *N*-glycans, in K562 cells.

## Discussion

**Figure 9:**
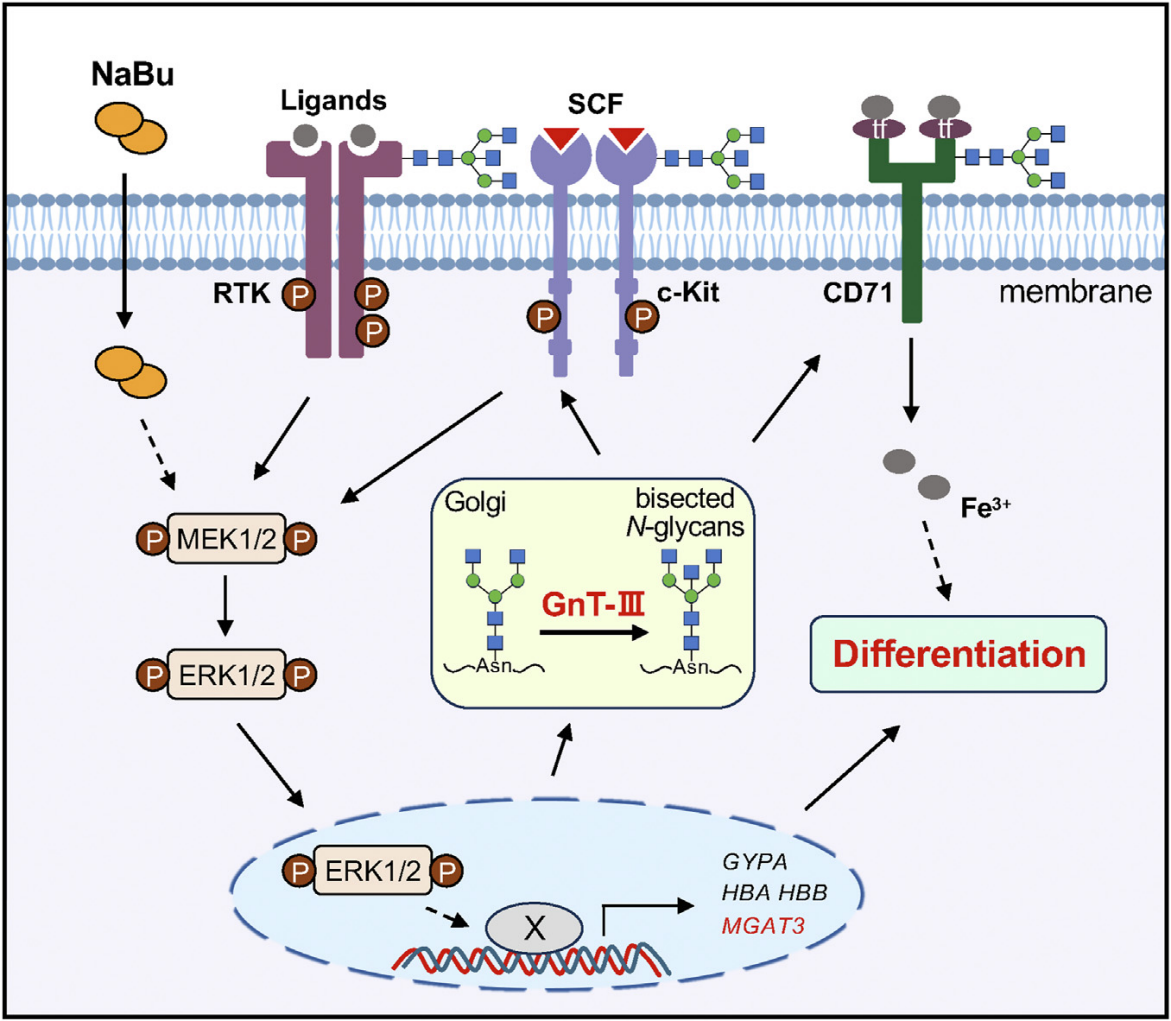
**Proposed molecular mechanism for the regulation of NaBu-induced erythroid differentiation by GnT-Ⅲ**. This study demonstrates that GnT-Ⅲ expression can regulate NaBu-induce erythroid differentiation through the RTKs/ERK1/2 pathway. GnT-Ⅲ modifies membrane glycoproteins such as c-Kit and CD71, which are critical for erythroid differentiation, enhancing their biological functions including protein stability and signal transduction. c-Kit, a potent receptor tyrosine kinase (RTK), is pivotal in erythroid differentiation (68), while CD71 facilitates the cellular internalization of iron ions from transferrin (69). Conversely, GnT-Ⅲ expression and bisected *N*-glycans are augmented *via* ERK1/2 activation during NaBu-induced erythroid differentiation. Inhibition of ERK1/2 activation suppresses GnT-Ⅲ expression and erythroid differentiation, suggesting a positive feedback loop between GnT-Ⅲ and ERK1/2 activation. Whether NaBu can directly activate MEK1/2 or P-ERK1/2 enters the nucleus to act directly as transcription factors or form a complex with others (X) for transcriptional regulation remains to be studied further. The *dotted line* (- - -) indicates unclear and/or indirect effects. Given the essential role of ERK1/2 signaling in NaBu-induced erythroid differentiation of K562 cells, manipulating GnT-Ⅲ could represent a novel approach for differentiation therapy. ERK, extracellular signal–regulated kinase; GnT, *N*-acetylglucosaminyltransferase; NaBu, sodium butyrate.

In the present study, we established an erythroid differentiation model using K562 cells induced by NaBu and identified four results: (1) the expression levels of MGAT3 and bisected N-glycans were greatly increased in the erythroid differentiation cells; (2) MGAT3 KO suppressed the induction of differentiation as validated by cell coloration, β-globin, CD235a expression, and the activation of the RTKs/ERK1/2 pathway; (3) inhibition of the ERK1/2 signaling impaired the NaBu-induced erythroid differentiation, accompanied by the decreased the expression levels of MGAT3 and bisected N-glycans; and (4) membrane proteins, such as c-Kit and CD71, which are closely related to erythroid differentiation, were modified by GnT-Ⅲ to regulate their biological functions. Therefore, we conclude that GnT-Ⅲ plays a pivotal role in erythroid differentiation *via* the ERK1/2 signaling pathway (Fig. 9). This study elucidates a novel mechanism underlying the involvement of GnT-Ⅲ in erythroid differentiation, providing new insights into the role of *N*-glycosylation in regulating cancer differentiation.

On the other hand, previous studies have shown that *Mgat3*-KO mice are viable and reproduced normally, without significant alterations in circulating leukocytes and erythrocytes compared to WT mice (36), which seems to contradict to the results observed in the present study. The different phenotypes could be speculated to arise from 1) the complexity of erythrocyte differentiation *in vivo*, which involves the coordination of a network of transcription factors and epigenetic regulators from surrounding cells; 2) the possibility that other pathways *in vivo* may compensate for *Mgat3*-KO–mediated deficiencies; and 3) the role of BCR-ABL, formed by a reciprocal chromosomal translocation to create an aberrant BCR-ABL gene on chromosome 22, which is critical to the pathogenesis of CML due to its effects on aberrant cell signaling. Coincidently, the human *MGAT3 is mapped* on chromosome 22, suggesting that the translocation of BCR-ABL may alter the functions of GnT-Ⅲ, as observed in this study. The precise mechanisms require further investigation.

Aberrant glycosylation of membrane or secreted proteins, associated with neoplastic transformation, is a prominent characteristic of cancer (37, 38). GnT-Ⅲ has been reported to be profoundly involved in cell adhesion, growth, invasion, differentiation, and cancer metastasis (22, 39, 40). For instance, EMT is crucial in cancer initiation and metastasis (41). TGF-β1–induced EMT downregulates GnT-Ⅲ expression, whereas overexpression of GnT-Ⅲ inversely suppresses EMT by prolonging E-cadherin turnover on the cell surface (23). GnT-Ⅲ negatively regulates cell adhesion and migration by modulating integrin family proteins such as α3β1 and α5β1 (42, 43). The molecular mechanism has been speculated that the modification by the bisecting GlcNAc on integrin may change its conformation and interrupt ligand binding (44). In fact, the crystal structure of integrin α5β1 has revealed that the interface between fibronectin and α5β1 is surrounded by some *N*-glycans, which may participate in the regulation of their interaction (45). Furthermore, GnT-Ⅲ specifically modifies site-4 from among 14 other potential *N*-glycosylation sites on integrin α5 (46). GnT-Ⅲ also selectively modifies specific target glycoproteins; for example, it modifies potential *N*-glycosylation sites on TNFR2 but not TNFR1, influencing downstream signaling pathways (3). Loss of bisecting GlcNAc on TNFR2 promotes auto-trimerization and up-regulates P-glycoprotein, promoting chemoresistance, which can be suppressed by GnT-Ⅲ overexpression. Here, we demonstrate that GnT-Ⅲ modifies c-Kit and CD71 membrane proteins, critical in erythroid differentiation. c-Kit–mediated cellular signaling and CD71 stability were significantly suppressed in *MGAT3* KO cells compared to WT cells, suggesting that GnT-Ⅲ expression profoundly impacts tumor biology, including malignancy and differentiation. Therefore, although the amount of bisected *N*-glycans is only minor among *N*-glycans (Fig. 3 and Table S2), they may specifically modify *N*-glycosylation sites on specific proteins, such as c-Kit, contributing significantly to cell differentiation.

GnT-Ⅲ expression is regulated by cell–cell adhesion, significantly up-regulated in epithelial cells expressing E-cadherin but not in E-cadherin–deficient cells (47, 48). *N*-glycosylation at Asn-633 of E-cadherin is essential for its expression, folding, and trafficking (49, 50). GnT-Ⅲ overexpression enhances E-cadherin retention at the cell border, enhancing E-cadherin–mediated homotypic adhesion, suggesting a positive feedback loop with E-cadherin expression (40). However, this does not apply to K562 cells, which do not express E-cadherin. β-catenin, a downstream factor of E-cadherin and a central player in the canonical Wnt signaling pathway, suppresses GnT-Ⅲ expression by binding to lymphoid enhancer factor-1/T-cell factor, thereby inhibiting *MGAT3* expression (23). Wnt signaling promotes tumor progression, potentially promoting cell proliferation and invasion rather than differentiation. Here, we demonstrate an alternative GnT-Ⅲ induction pathway *via* NaBu-induced ERK/MAPK signaling, enhancing GnT-Ⅲ expression and erythroid differentiation. Conversely, ERK/MAPK signaling inhibition suppresses differentiation and *MGAT3* expression (Fig. 8). *MGAT3* KO significantly inhibited NaBu-induced erythroid differentiation while enhancing cell proliferation (Fig. 5), indicating a positive feedback loop between cell differentiation and GnT-Ⅲ expression (Fig. 9). In addition, we cannot exclude the influences from an interplay between glycan and redox (51). Glycan synthesis occurs in the endoplasmic reticulum and Golgi, so it is reasonable to assume that redox changes in these compartments affect glycosylation reactions, especially in erythroid differentiation, warranting further study.

CML is characterized by abnormal hematopoietic cell proliferation in the bone marrow. Unfortunately, many patients develop drug resistance or experience severe side effects from the targeted therapy, tyrosine kinase inhibitor (8). Differentiation therapy offers a less toxic approach, using drugs to induce cancer cell differentiation, reducing proliferative capacity, and promoting terminal differentiation or apoptosis (52). For example, all-trans retinoic acid treats APL by inducing leukemia cell differentiation, improving patient outcomes (53). We found that inhibiting fucosylation enhances all-trans retinoic acid–induced differentiation in APL cells (54). Together, these findings, including ours, further suggest glycosylation's role in cell functions and differentiation.

A deeper understanding of differentiation mechanisms may lead to new therapeutic targets. The ERK/MAPK pathway, crucial in cell proliferation, apoptosis, and differentiation (55), is often hyperactive in various cancers (56). GnT-Ⅲ overexpression enhances ERK activation in several cancer cell lines, modifying RTK receptors such as EGFR and c-Met to enhance downstream signaling (57, 58). However, GnT-Ⅲ can also downregulate RTK receptors like TrKA and EGFR-mediated signals in PC12 cells (59, 60) and other cells (61). Thus far, these discrepancies remain unclear, but we can speculate that it is due to different target proteins and/or cell lines. Here, we show that GnT-Ⅲ modifies c-Kit, a potential RTK receptor in erythroid differentiation, suppressing its mediated ERK activation in *MGAT3* KO cells (Fig. 6), suggesting that GnT-Ⅲ expression upregulates the ERK activation. Inhibiting ERK activation effectively blocks NaBu-induced erythroid differentiation. Recently, Feng J. *et al*. reported lower GnT-Ⅲ levels and bisecting GlcNAc on bone marrow stroma in myelodysplastic syndrome and acute myeloid leukemia patients compared to healthy donors (62), further suggesting GnT-Ⅲ as a potential target associated with the ERK pathway in differentiation therapy.

## Experimental procedures

### Antibodies and reagents

The experiments were performed using the following antibodies and reagents: Antibodies against CD71 (13113), c-Kit (3074), p44/42 MAPK (ERK1/2; 9102), phospho-p44/42 MAPK (Thr202/Tyr204) (p-ERK1/2; 4370), SAPK/JNK (9252), phospho-SAPK/JNK (Thr183/Tyr185) (4668), p38 MAPK (9212), phospho-p38 MAPK (Thr180/Tyr182) (4511), the peroxidase-conjugated secondary antibody against rabbit (7074S), and U0126 (9903) were purchased from Cell Signaling Technology; biotinylated E4-PHA (B-1385), DSA (B-1185), biotinylated L4-PHA (B-1115–2), biotinylated SNA (B-1305), biotinylated Wheat germ agglutinin (B-1025-5), and ABC kit (PK-4000) were from Vector Laboratories; biotinylated LCA (J207) and E4-PHA agarose (J311) were obtained from J-Oil Mills; biotinylated MAM lectin (#BA-s7801-2) was purchased from EY Laboratories; The anti-α-Tubulin (T6199) antibody and peroxidase-conjugated secondary agents against mouse (AP124P), NaBu (303410), SAHA (HY-10221), and hemin (HY-19424) were acquired from Sigma; The antibody against CD235a (JC159) was from Thermo Fisher Scientific; The goat anti-mouse IgG Alexa Fluor 568, goat anti-rabbit IgG Alexa Fluor 488 and streptavidin conjugate Alexa Fluor 647 antibody were from Invitrogen; phosphotyrosine (p-Tyr) antibody (sc-7020) was from Santa Cruz; PNGase F was from Roche. The cycloheximide (037-20991) was from Wako; CCK-8 was obtained from DOJINDO (341-07761).

### Cell culture and induction of erythroid differentiation

The human leukemia cell line K562 was obtained from the Cell Resource Center for Biomedical Research, Tohoku University. The HEL cells were purchased from RIKEN Cell Bank. The cells were cultured in RPMI 1640 medium supplemented with 10% fetal bovine serum (endotoxin level 10 EU/ml/ml) and 1% antibiotic/antimycotic agent (Gibco) in a 5% CO_2_, 37 °C humidified atmosphere. During erythroid differentiation, K562 cells at a density of 1 × 10^5^ cells/ml were treated with NaBu at different concentrations over a period of 4 days. In addition, the K562 cells and HEL cells were treated with 10 μM hemin for 6 days. These cells were confirmed to be free from *mycoplasma* using the e-Myco *Mycoplasma* PCR Detection kit (iNtRON Biotechnology, Republic of Korea).

### Pull-down assay, Western blot, and lectin blot

Cells were washed three times with ice-cold PBS and subsequently lysed in cell lysis buffer (comprising 20 mM Tris–HCl, pH 7.4, 150 mM NaCl, 1% Triton X-100) containing protease and phosphatase inhibitors (Nacalai Tesque) on ice for 30 min. The lysates were then centrifugated at 15,000 *g* and 4 °C for 10 min. The supernatant was immediately transferred to a fresh centrifuge tube, and the pellet was discarded. Protein concentration in the supernatant was quantified using a bicinchoninic acid protein assay kit (Pierce).

For the pull-down assay, equal quantities of proteins from each cell lysate were incubated with PHA-E4-Agarose at 4 °C overnight with rotation. Subsequently, the precipitates were washed twice with tris-buffered saline and detected by Western blotting using specific antibodies as indicated.

Western blot and lectin blot were performed as follows: equal amounts of protein (10 ug) were separated on 7.5% or 15% SDS/PAGE gels at 100 V and transferred to polyvinylidene difluoride membranes (Millipore Sigma) at 10 V for 1 h. These membranes were blocked with freshly prepared 5% bovine serum albumin (BSA) for lectin blotting or 5% nonfat dried milk for Western blotting, supplemented with 0.05% Tween-20, at room temperature for 90 min. Subsequently, the membranes were incubated with specific primary antibodies or biotinylated lectins at 4 °C overnight. After washing three times for 5 min each, the membranes were incubated with appropriate secondary antibodies. Immunoreactive bands were detected using an immobilon Western Chemiluminescent horseradish peroxidase Substrate (Millipore) according to the manufacturer's instructions.

### Establishment of *MGAT3*-KO cells

The pSpCas9(BB)-2A-GFP (PX458) plasmid was obtained from Addgene (PX458: Addgene #48138). *MGAT3* KO cells were generated using guide RNA (5′- CATGCGCAAGTCGCTCTACG -3′) targeted to human GnT-Ⅲ genes, which are adjacent to Cas9 in the pSpCas9(BB)-2A-GFP vector. GFP-expressing cells were sorted to select for successful transfection. The stable K562-*MGAT3* KO cell line was established by electroporating cells following the manufacturer's recommendations (Amaxa cell line Nucleofector kit; Lonza). After 24 h, cells showing positive fluorescence were sorted using FACSAria II (BD Bioscience), diluted, and seeded on 96-well plates to obtain single clones. These single clones were expanded over a 4-week period. Genomic DNA was extracted from these clones, and the CRISPR target region was validated by PCR amplification using the following primers: Forward primer, 5′- TTCCTCACCCAGGACGGC -3′; Reverse primer, 5′- CGAGCTTGAAGTAGATGCCCT-3′. Sequencing was performed using the reverse primer to confirm the desired genomic modifications.

### Real-time PCR qPCR

RNAs were extracted using TRI Reagent (Molecular Research Center, Lnc), and then 1 μg of total RNAs was reverse transcribed into complementary DNA using a PrimeScript RT reagent with a genomic DNA Eraser (Takara, Japan) according to the manufacturer's instructions. The specific primer sequences are listed in Table 1. PCR products were diluted to 50 ng/μl and analyzed using StepOnePlus (Applied Biosystem). Real-time PCR analyses were performed using TB Green Premix Ex Taq II (Tli RNaseH Plus) (Takara) under the following conditions: initial denaturation at 95 °C for 30 s, followed by 40 cycles of denaturation at 95 °C for 5 s, and annealing and extension at 60 °C for 30 s.

**Table 1:** Primer sequences for real-time PCR

Target genes	Primer sequences (5′-3′)	
	Forward sequences	Reverse sequences
HBA	CCAAGACCTACTTCCCGCAC	CGTTGGGCATGTCGTCCA
HBB	TCCTGATGCTGTTATGGGCA	CTCACTCAGTGTGGCAAAGGT
GYPA	TGATACGCACAAACGGGACA	ACCAGCCATCACCCCAAAAA
TFRC	TCGGAGAAACTGGACAGCAC	ATCACGCCAGACTTTGCTGA
MGAT1	TGACCAGCACCTCAAGTTTATC	CGGAACTGGAAGGTGACAATAC
MGAT2	AGAGTGCCCTGAATGTGATG	CACAGTCTCCAGCATGAAAGA
MGAT3	GCCGCGTCATCAACGCCATCAA	CAGGTAGTCGTCGGCGATCCA
MGAT4A	GGCTATCACACCGATAGCTGGAG	TCCACCATTCCTTCTGCAACACC
MGAT4B	ACAACCCTCAGTCAGACAAGGAGG	GGTACCCTCAGAAGCCCGCAGCTT
MGAT5	GACCTGCAGTTCCTTCTTCG	CCATGGCAGAAGTCCTGTTT
FUT8	GACAGAACTGGTTCAGCGGAGA	GCAGTAGACCACATGATGGAGC
GAPDH	ACTCCACTCACGGCAAATTC	CCCTGTTGCTGTAGCCGTAT

### Flow cytometry

Cells (1 × 10^6^) were harvested and washed with ice-cold PBS. Equal amounts of cells were then incubated with biotinylated lectins (E4-PHA) or antibodies against CD235a in magnetic-activated cell sorting buffer (0.1% BSA in PBS) for 30 min on ice. After incubation, the cells were further incubated with streptavidin conjugate Alexa Fluor 647 (1:3000) or goat anti-mouse IgG Alexa Fluor 568 (1:500) for 30 min at room temperature in the dark. Subsequently, the cells were washed and resuspended in 800 μl of magnetic-activated cell sorting buffer. Fluorescence intensities were detected using the Attune flow cytometer (BD Biosciences) following flow cytometry experiment standard (63) and analyzed using FlowJo software (https://www.flowjo.com).

### Immunofluorescence staining

Cells (1 × 10^6^) were collected into 1.5 ml tubes. After washing twice with PBS, the cells were fixed with 4% paraformaldehyde for 30 min. Subsequently, the cells were treated with 0.1% Triton X-100 in PBS for 10 min, followed by incubation with 5% BSA in PBS at room temperature for 2 h to block nonspecific staining. The cells were then stained with an antibody against CD71 (green) at a dilution of 1:100 at 4 °C overnight. Meanwhile, the plasma membrane was stained using Wheat germ agglutinin lectin (red) at a dilution of 1:300. After washing with PBS, the cells were incubated with secondary antibodies (goat anti-rabbit IgG Alexa Fluor 488 and streptavidin conjugate Alexa Fluor 647) for 2 h. Additionally, the cells were stained with 4′,6-diamidino-2-phenylindole at room temperature for 8 min in the dark. Detection was accomplished using a ZEISS LSM 900 confocal microscope.

### *N*-glycan analysis of cell membrane proteins *via* MALDI-TOF MS analysis

For *N*-glycan analyses, cell pellets were prepared by homogenate and separated by ethanol precipitation, following previously described methods (64). *N*-glycans were extracted from protein pellets through a series of steps, including reduction, alkylation, trypsin digestion, and deglycosylation using PNGase F (65). The released *N*-glycans were subjected to a glycoblotting procedure in combination with the aminolysis-sialic acid linkage specific alkylamidation method, as outlined in previous studies (66, 67). Subsequently, purified and labeled *N*-glycans were analyzed by MALDI-TOF MS analysis, following established protocols (66).

### LC-ESI-MS/MS analysis of bisected *N*-glycans

Hydrophilic interaction chromatography-purified *N*-glycans from WT/NaBu cells were dissolved in 2% acetonitrile (MeCN) containing 0.1% formic acid (FA) at a concentration of 0.5 μg proteins/μl and subjected to LC-ESI-MS/MS analysis. The analysis of bisected *N*-glycans was performed on an UltiMate 3000 RSLCnano system (Thermo Fisher Scientific) coupled to a Q Exactive mass spectrometer (Thermo Fisher Scientific) equipped with a nano ESI source. The LC system had a trap column (C18 PepMap 100, 0.3 × 5 mm, 5 μm, Thermo Fisher Scientific) and an analytical column (NTCC-360/75–3-125, Nikkyo Technos). After loading the sample onto a trap column, the valve was switched to allow the separation of *N*-glycans using the analytical column. Separation was carried out with a 30-min gradient of water containing 0.1% FA (mobile phase A) and MeCN containing 0.1% FA (mobile phase B) at a flow rate of 300 nl/min. The gradient was performed as follows: 0 to 3 min, 2% B; 3 to 33 min, 2% to 40% B; 33 to 35 min, 40% to 95% B; 35 to 45 min, 95% B; 45 to 47 min, 95%–2% B; and 47 to 60 min, 2% B. MS/MS spectra were obtained using targeted selected ion monitoring/data-dependent–MS/MS acquisition mode. Parameters for selected ion monitoring are as follows: polarity, positive; resolution, 70,000; automatic gain control (AGC) target, 5e4; maximum ion trap, 100 msec; and isolation window, 4.0 *m/z*. Parameters for data-dependent–MS/MS are as follows: resolution, 35,000; AGC target, 2e5; maximum ion trap, 100 msec; isolation window, 1.6 *m/z*; normalized collision energy, 15; minimum AGC target, 8e3; intensity threshold, 8e4; and dynamic exclusion, 0.2 s.

### Cell proliferation assay

Equal amounts of cells (2 × 10^3^ cells/100 μl/well) were plated into a 96-well plate and cultured in normal media with or without NaBu at 1 mM. The cells were then incubated for various durations (0, 24, 48, 72, and 96 h). After the respective incubation periods, 10 μl of CCK-8 solution was added to each well and further incubated for another 1 h at 37 °C. Subsequently, cell viability was assessed by measuring the absorbance at 450 nm using a microplate reader (SYNERGY multimode reader; BioTek).

### Statistical analysis

All data are presented as the mean ± SD obtained from at least three independent experiments. Statistics analyses were performed using a one-way ANOVA with Tukey's *post hoc* test, two-way ANOVA with Tukey's multiple comparisons test or an unpaired Student *t* test using GraphPad Prism 6.0 software (GraphPad Software, Inc). A probability value of *p* was considered as follows: ns (no significance) for *p* > 0.05; ∗*p* < 0.05; ∗∗*p* < 0.01; and ∗∗∗*p* < 0.001.

## Data availability

All data presented in the figures, tables, and supplementary information of this paper are available. The Glycoproteomic raw MS data and the identification result file for analysis of glycan structures on peptides have been deposited at the GlycoPOST (announced ID: GPST000415; https://glycopost.glycosmos.org/preview/1024471763666be582292fb; PIN CODE:1143).

## Supporting information

This article contains supporting information.

## Conflicts of interest

The authors declare that they have no conflicts of interest with the contents of this article.
