# Supplementary material for: The acetylglucosaminyltransferase GnT-Ⅲ regulates erythroid differentiation through ERK/MAPK signaling
Source: J Biol Chem. 2024 Nov 19;300(12):108010. doi: 10.1016/j.jbc.2024.108010 (PMC11699732; doi:10.1016/j.jbc.2024.108010)
Supplement: Supplemental Figs. S1–S5 [file mmc1.pdf]

Supplemental Fig. 1

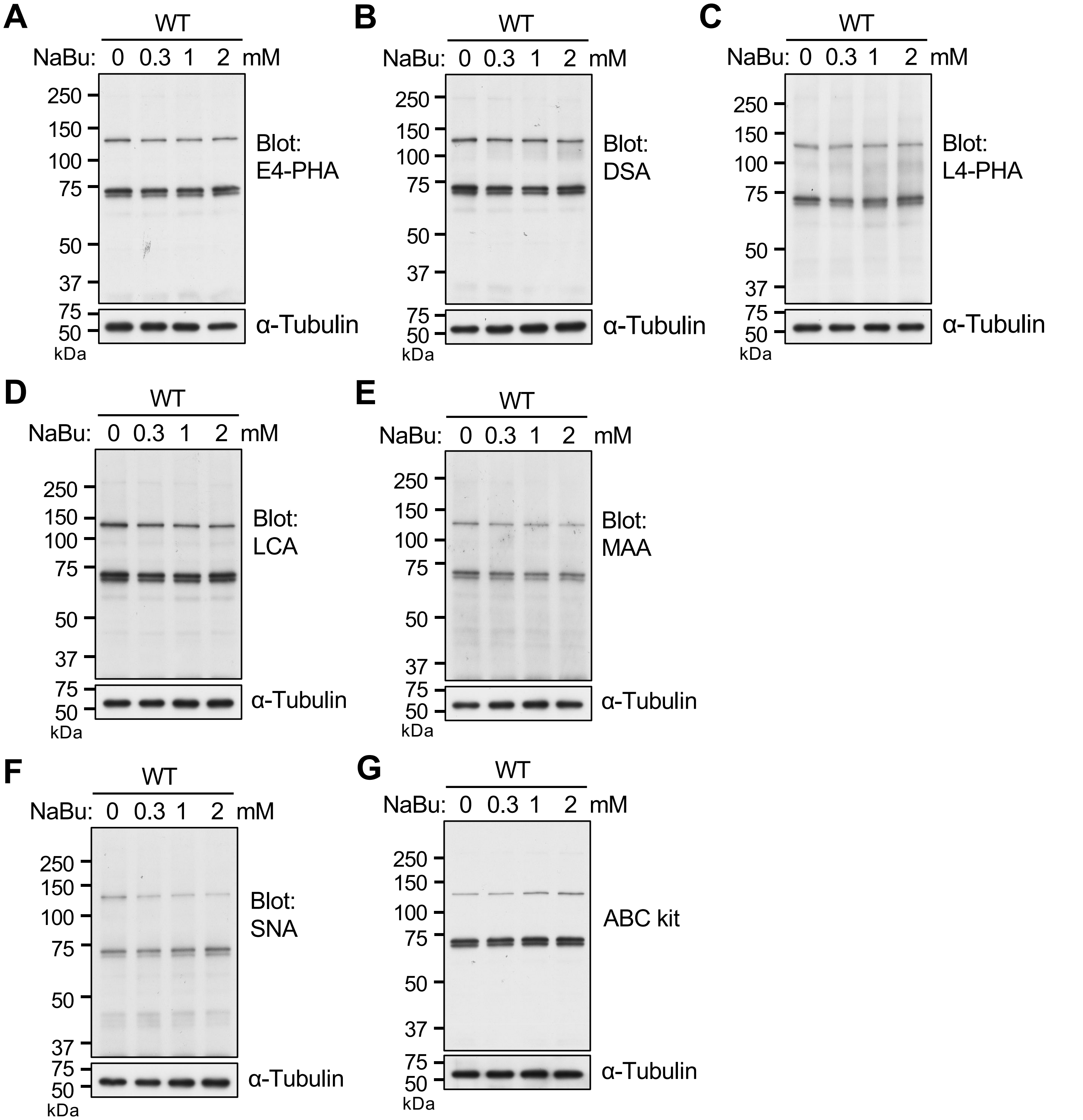

sFig.1. Lectin blot analysis of cell lysates treated with PNGase F.

A-F, equal amounts of cell lysates were treated with PNGase F to remove *N*-linked glycans. The samples were then subjected to lectin blotting using biotinylated lectins. **G**, cell lysates were directly stained with ABC kit without lectin. α-Tubulin was used as a loading control.

Supplemental Fig. 2

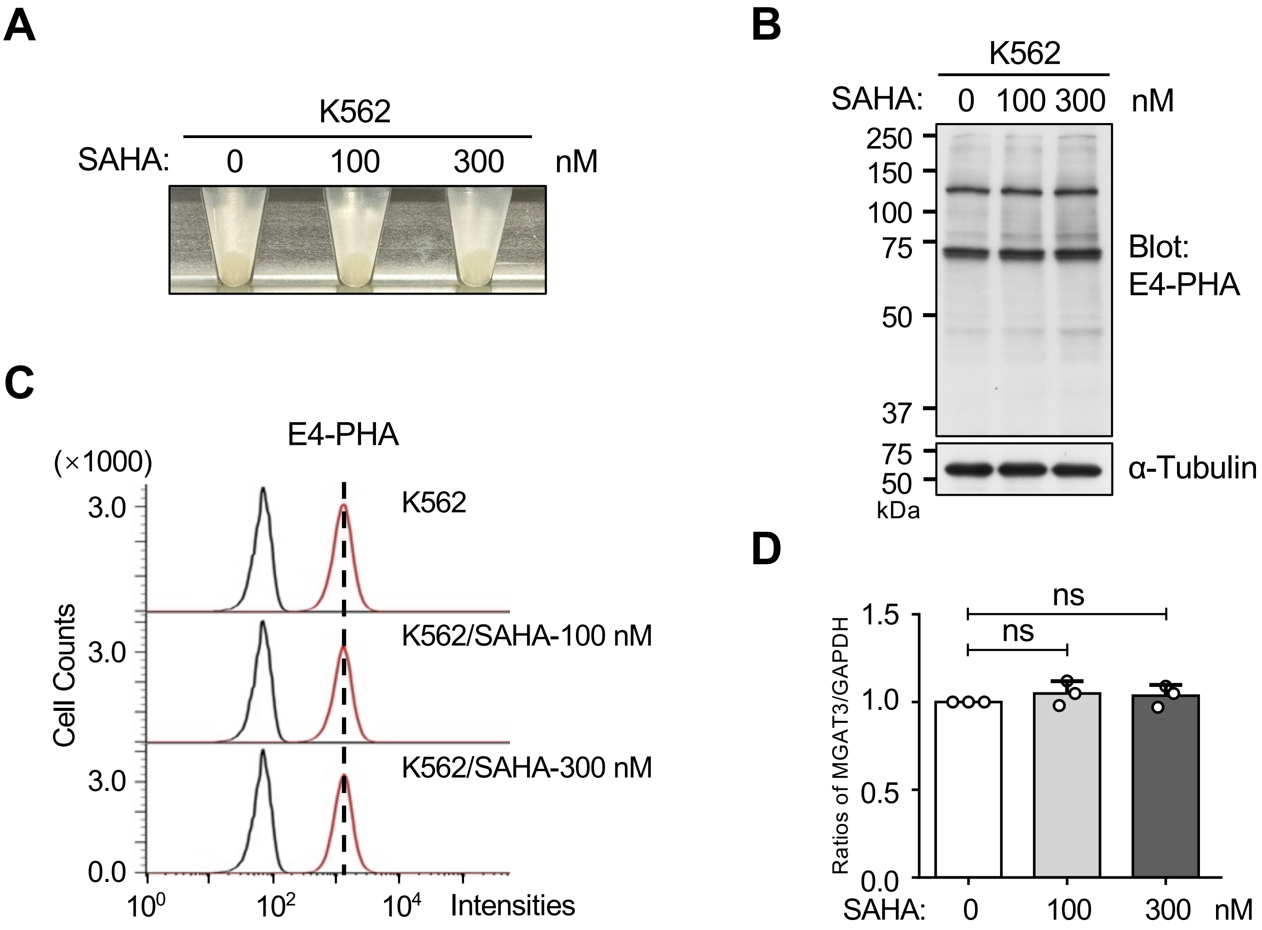

sFig.2. Effects of suberanilohydroxamic acid (SAHA) on the expression of bisected *N*-glycans and *MGAT3*.

A, K562 cells were treated with SAHA at 100 nM and 300 nM for 4 days to observe changes in cell color. **B,** equal amounts of protein from these cells were loaded onto a 7.5% SDS-PAGE gel to compare the expression of bisected *N*-glycans using E4-PHA lectin, with α-tubulin serving as a loading control. **C,** the levels of bisected *N*-glycans on the cell surface were detected by flow cytometry using E4-PHA lectin. **D,** mRNA expression level of GnT-Ⅲ was determined using qPCR. GAPDH served as the internal control. All values were normalized to the GAPDH levels, with the ratio of WT without SAHA set as 1.0. Data are presented as mean ± SD from three independent experiments, analyzed using one-way ANOVA with Tukey's post hoc analysis. ns, no significance.

Supplemental Fig. 3

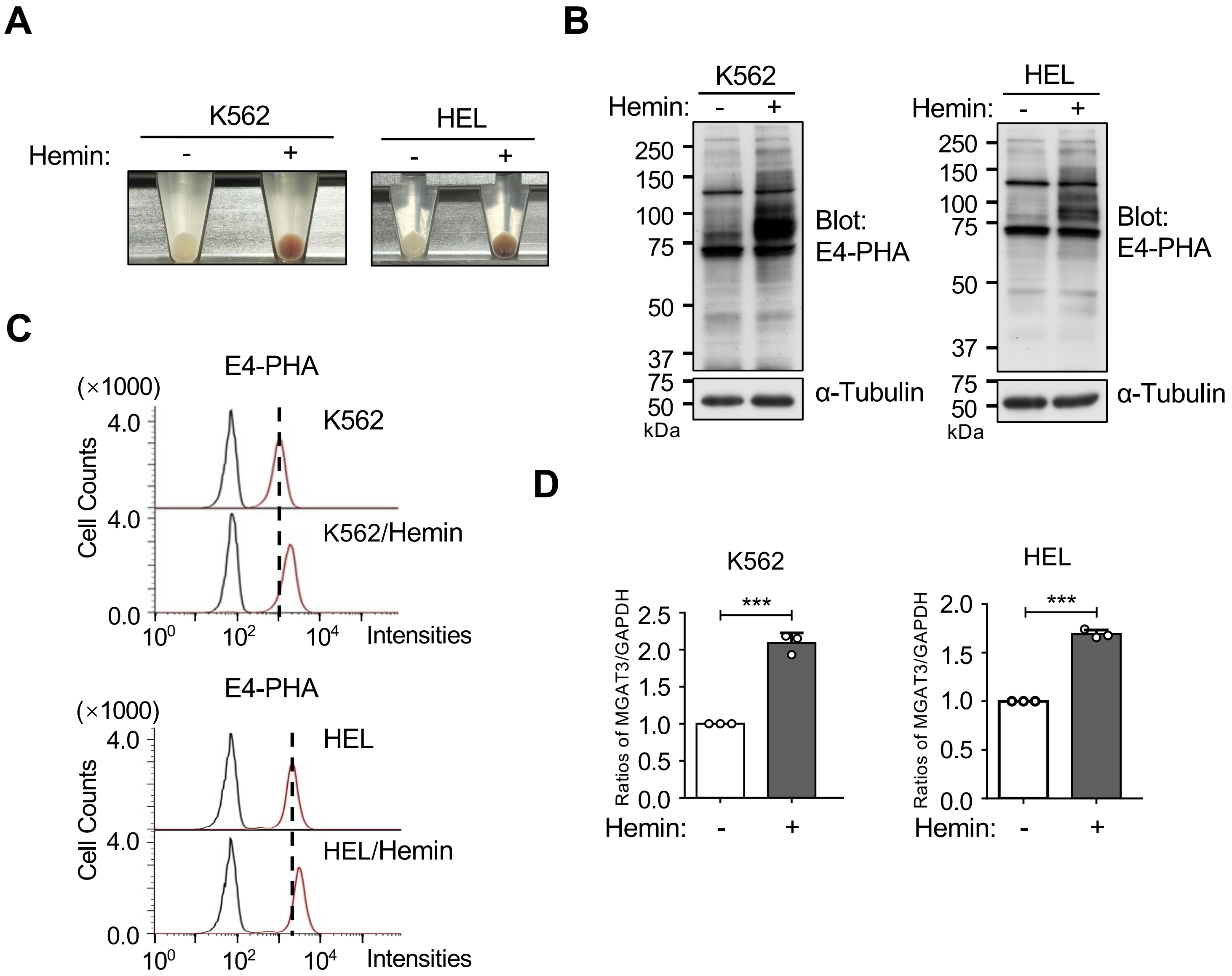

sFig.3. Levels of bisected *N*-glycans were detected during hemin-induced erythroid differentiation in K562 and HEL cells.

A, K562, and HEL cells underwent erythroid differentiation following treatment with 10 µM hemin for 6 days, resulting in a reddish appearance in the treated cells. **B,** cell lysates were extracted from WT and hemin-induced K562 and HEL cells. Equal amounts of cell lysates were loaded onto a 7.5% SDS-PAGE gel to detect bisecting GlcNAc levels via lectin blotting with E4-PHA. α-Tubulin was used as a loading control. **C,** the levels of bisected *N*-glycans on the cell surface of K562 and HEL cells were assessed by flow cytometry using E4-PHA lectin. **D,** mRNA levels of GnT-Ⅲ in K562 and HEL cells were determined using qPCR, with GAPDH as the internal control. Values were normalized to WT cells without hemin, which was set as 1.0. Statistical significance was assessed using the unpaired Student's t-test, with p-values indicated as ***$p < 0.001$.

Supplemental Fig. 4

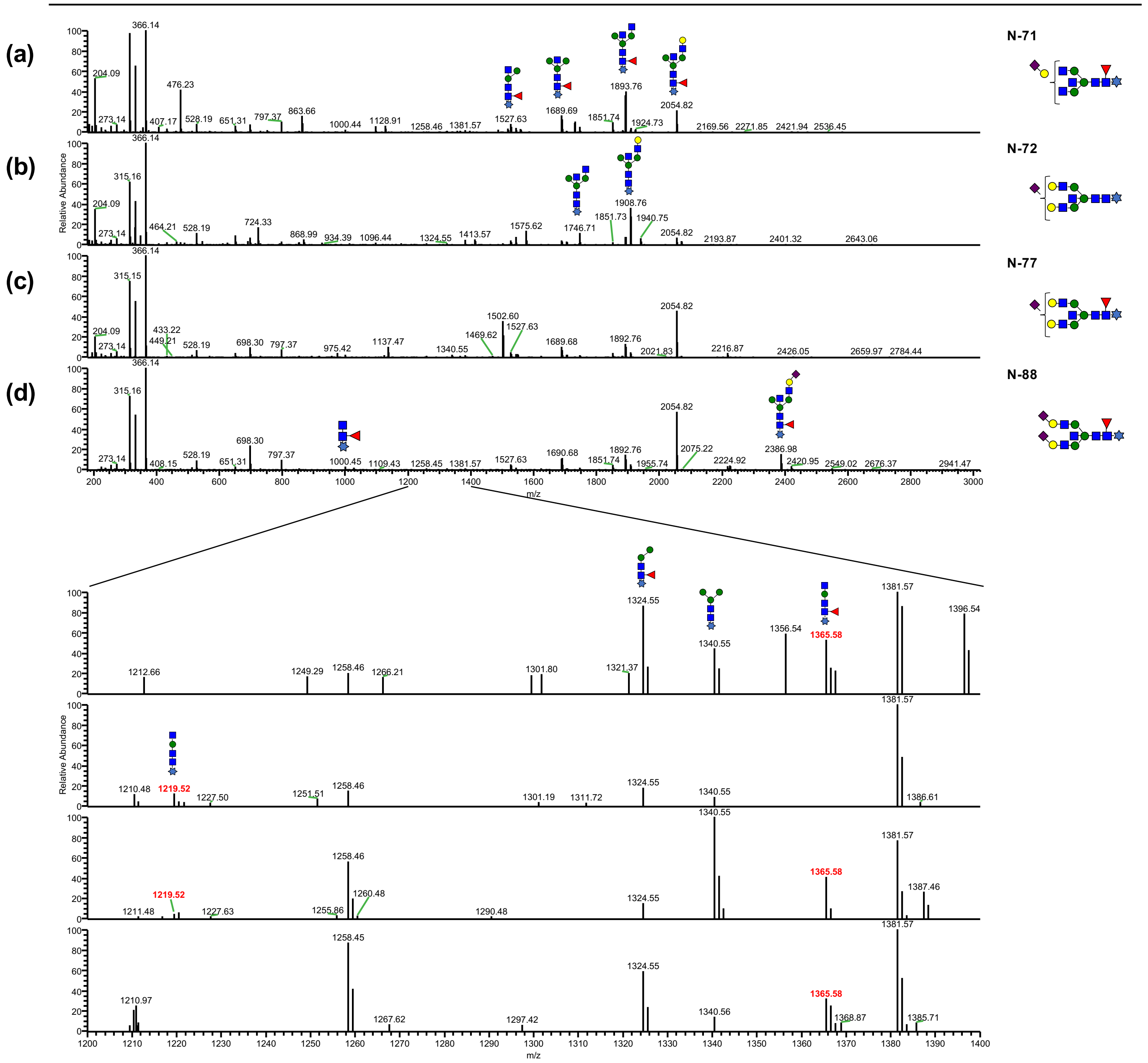

sFig.4. MS/MS spectra and expected structure of bisected *N*-glycans.
A, Glycan #***N*-71**, *m*/*z* 864.02, *z* = +3. **B,** Glycan #***N*-72**, *m*/*z* 869.35, *z* = +3. **C,** Glycan #***N*-77**, *m*/*z* 918.04, *z* = +3. **D,** Glycan #***N*-88**, *m*/*z* 1028.76, *z* = +3.

Supplemental Fig. 5

A

gRNA

WT: AGGGTGCCCGGCTGCTTCCAGAAGAAGCCGTAGAGCGACTTGCGCATGTGGAAGGC

KO-1 allele-1: AGGGTG--GAAGGC

KO-1 allele-2: AGGGTGCCCGGCTGCTTCCAGAAG---------------------------GCGAAGGGC

KO-2 allele-1/2: AGGGTGCCCGGCTGCTTCCAGAAGAAGCCGTAGAGCGACTTGCGCATGTGGAAGGC

GAA C

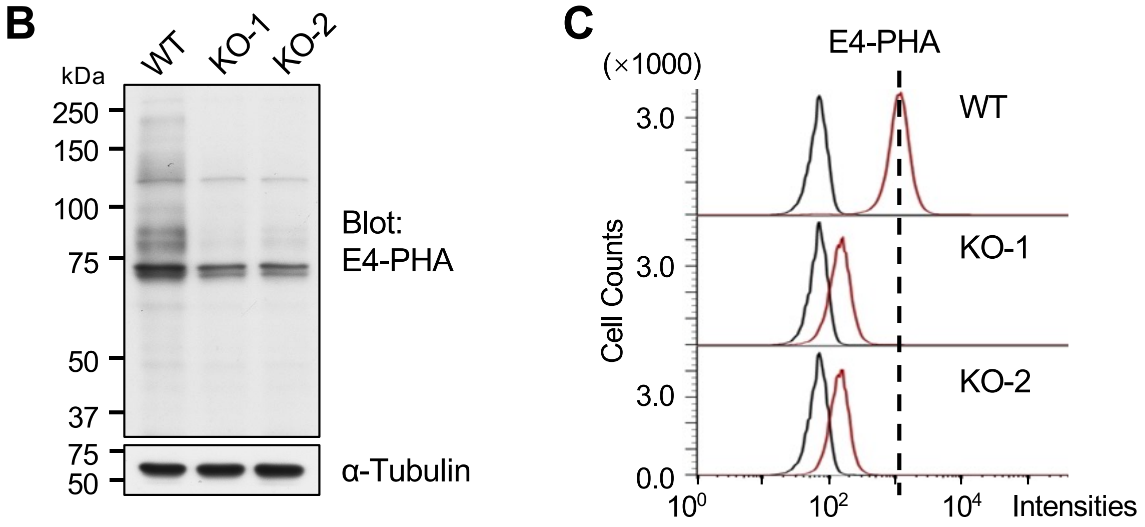

sFig.5. Establishment of *MGAT3* KO K562 cell line

A, the *MGAT3*-targeting gRNA was designed (underlined). Compared to WT cells, the sequence of *MGAT3* KO-1 cells showed 44 bases deletion in allele-1 and 23 bases deletion in allele-2, and 4 bases insertion mutation (GAA inserted between A and G; C inserted between C and A) in the KO-2 K562 cells. **B,** cell lysates were extracted from WT K562 cells and two *MGAT3* KO cells (KO-1 and KO-2). Equal amounts of cell lysates were loaded into 7.5% SDS-PAGE gel to detect bisecting GlcNAc levels by lectin blotting with E4-PHA. α-Tubulin was used as a loading control. **C,** expression levels of bisected *N*-glycans on the cell surface were detected by flow cytometry using E4-PHA lectin.
